# Effect of growth rate on transcriptomic responses to immune stimulation in wild-type, domesticated, and GH-transgenic coho salmon

**DOI:** 10.1186/s12864-019-6408-4

**Published:** 2019-12-27

**Authors:** Jin-Hyoung Kim, Daniel J. Macqueen, James R. Winton, John D. Hansen, Hyun Park, Robert H. Devlin

**Affiliations:** 1 Fisheries and Oceans Canada, 4160 Marine Drive, West Vancouver, BC V7V 1N6 Canada; 2 The Roslin Institute and Royal (Dick) School of Veterinary Studies, The University of Edinburgh, Midlothian, EH25 9RG UK; 3 US Geological Survey, Western Fisheries Research Center, 6505 NE 65th Street, Seattle, 98115 USA; 4 Present Address: Korea Polar Research Institute, Unit of Polar Genomics, 26 Sondomirae-ro, Yeonsu-gu, Incheon, 21990 Republic of Korea; 5 Divison of Biotechnology, College of Life Sciences and Biotechnology, Korea University, Seoul, 02841 Republic of Korea

**Keywords:** Growth, Immunity, Transgenesis, Selective breeding, Pleiotropy, Growth hormone, Coho salmon, Transcriptomics, Poly I:C, PGN

## Abstract

**Background:**

Transcriptomic responses to immune stimulation were investigated in coho salmon (*Oncorhynchus kisutch*) with distinct growth phenotypes. Wild-type fish were contrasted to strains with accelerated growth arising either from selective breeding (i.e. domestication) or genetic modification. Such distinct routes to accelerated growth may have unique implications for relationships and/or trade-offs between growth and immune function.

**Results:**

RNA-Seq was performed on liver and head kidney in four ‘growth response groups’ injected with polyinosinic-polycytidylic acid (Poly I:C; viral mimic), peptidoglycan (PGN; bacterial mimic) or PBS (control). These groups were: 1) ‘W’: wild-type, 2) ‘TF’: growth hormone (GH) transgenic salmon with ~ 3-fold higher growth-rate than W, 3) ‘TR’: GH transgenic fish ration restricted to possess a growth-rate equal to W, and 4) ‘D’: domesticated non-transgenic fish showing growth-rate intermediate to W and TF. D and TF showed a higher similarity in transcriptomic response compared to W and TR. Several immune genes showed constitutive expression differences among growth response groups, including perforin 1 and C-C motif chemokine 19-like. Among the affected immune pathways, most were up-regulated by Poly I:C and PGN. In response to PGN, the c-type lectin receptor signalling pathway responded uniquely in TF and TR. In response to stimulation with both immune mimics, TR responded more strongly than other groups. Further, group-specific pathway responses to PGN stimulation included NOD-like receptor signalling in W and platelet activation in TR. TF consistently showed the most attenuated immune response relative to W, and more DEGs were apparent in TR than TF and D relative to W, suggesting that a non-satiating ration coupled with elevated circulating GH levels may cause TR to possess enhanced immune capabilities. Alternatively, TF and D salmon are prevented from acquiring the same level of immune response as TR due to direction of energy to high overall somatic growth. Further study of the effects of ration restriction in growth-modified fishes is warranted.

**Conclusions:**

These findings improve our understanding of the pleiotropic effects of growth modification on the immunological responses of fish, revealing unique immune pathway responses depending on the mechanism of growth acceleration and nutritional availability.

## Background

Fish health is a critical factor determining the success of aquaculture [1] and survival of wild fish. In culture, fish health depends on external variables that can be largely controlled, for example, water and feed quality, husbandry stress and pathogen exposure. However, intrinsic factors, underpinned by genetics, are also central to fish health, including the status of systems controlling growth, nutrition and immunity, which are major targets for selective breeding to enhance growth rate and disease resistance [2–4]. Selective breeding has been exploited extensively in salmonid aquaculture and resulted in significant gains in target traits [5–9]. While such changes are of great benefit within the aquaculture sector, there exist ongoing concerns about the potential for escaped selectively-bred and domesticated fish to breed with wild populations and reduce their fitness by disrupting naturally-adapted genomes through introgression and hybridization. The genetic and physiological mechanisms causing phenotypic and fitness changes in different salmonid genotypes is understood at a basic level [8, 10, 11], but remains of great interest in the context of selective breeding and for understanding ecological impacts resulting from the interaction of wild and farmed fish.

Genetic engineering approaches, including transgenesis, provide an alternative to selective breeding for modification of traits of value within aquaculture. Selective breeding classically targets phenotypic variation without knowledge of the specific underlying genetic variation but rather alters the frequency of many alleles, including variants unrelated to the trait of interest. In contrast, transgenesis typically alters the expression of a single target gene of known major effect. In salmonids and other farmed fish species, emphasis has been placed on the achievement of high growth rates through the transgenic overexpression of growth hormone (GH) [8, 12–14]. GH transgenic fish possess modified gene expression, physiology and behaviour, including elevated appetite, enhanced feeding motivation, elevated feed conversion efficiency, elevated metabolic rate, and, in some cases, altered susceptibility to pathogens [14–19]. Despite a considerable body of literature comparing the characteristics of GH transgenic versus wild-type and growth-enhanced domesticated phenotypes, to date, the contrasting effect of GH and selection for high growth on the immune system remains poorly characterized. In coho salmon, past work showed that GH transgenesis alters immune phenotypic characteristics, with negative associated impacts on disease resistance [19, 20]. Moreover, a recent study highlighted a substantial attenuation of host defence gene responses to immune stimulation in skeletal muscle of GH transgenic salmon, altering downstream regulation of master growth controlling pathways dependent on GH and its impact on growth rate [21]. However, there are no published studies of the impact of immune stimulation on key tissues for host defence in growth-accelerated transgenic fish strains.

The objective of the present study was to improve our understanding of immune system function in wild-type versus growth-accelerated salmon strains achieved by GH transgenesis or selective breeding following domestication. This was achieved using RNA-Seq to characterize the transcriptomic responses of liver and head kidney, each a key immune tissues, to mimics of viral and bacterial infection under common garden conditions. The study revealed complex responses to immune stimulation that also differed among salmon strains with different growth rates and between tissue types. The data reported have importance for future considerations surrounding the applications of transgenesis in aquaculture, the evaluation of domesticated strains, and for risk assessments on the potential consequences of transgenic fish entering natural environments.

## Results

### Comparison of the growth response group transcriptomes

**Fig. 1:**
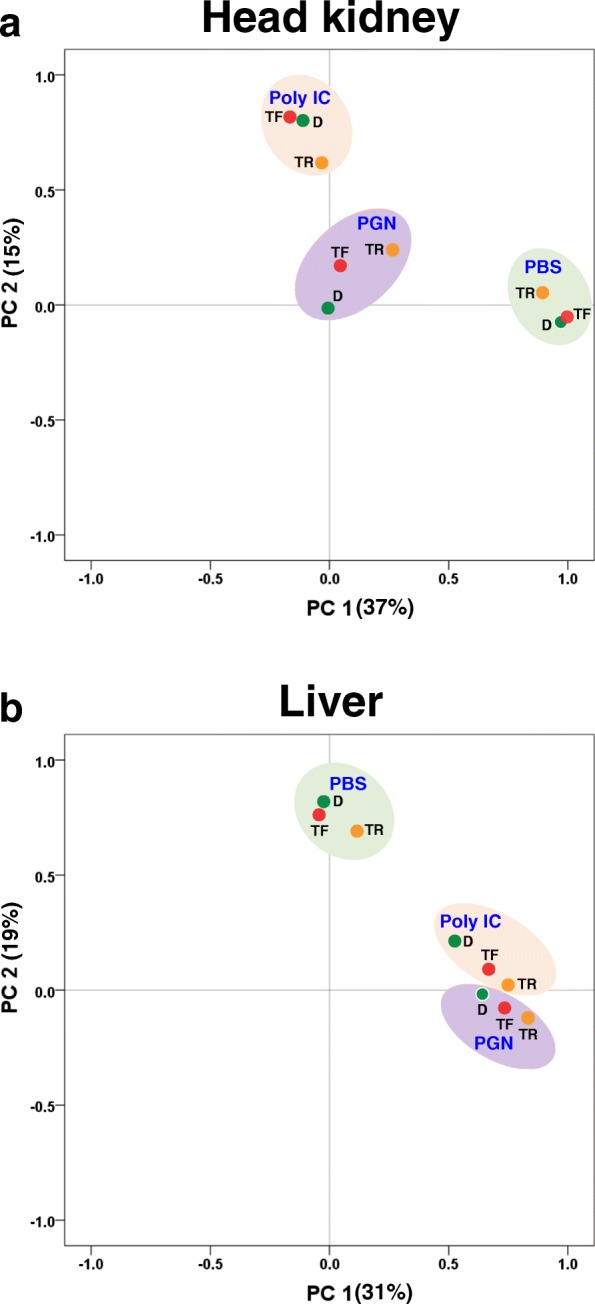
Principal component analysis (PCA) of all reads from RNA-Seq analysis for **a**) Head kidney and **b**) Liver treated with PBS, PGN and Poly I:C for. PBS, phosphate-buffered saline; PGN, peptidoglycan; Poly I:C, polyinosinic-polycytidylic acid. W, non-transgenic (wild-type) coho salmon on a full satiation ration; TF, GH transgenic coho salmon on a full satiation ration; TR, GH transgenic coho salmon on restricted ration equal to that consumed by W; D, domesticated coho salmon on a full satiation ration

The overall relative transcriptomic responses (compared to W fish) of the three growth groups (GH transgenic, TF; GH transgenic fed W satiating ration, TR; Domesticated, D) to immune stimulation are summarized by principal component analyses (PCA) (Fig. 1). Separate PCA plots were generated for the first two principle components for head kidney and liver treated with the immune stimulants (Poly I:C or PGN). For head kidney treated with PBS or Poly I:C, TF and D were clustered closely compared to TR (Fig. 1a), whereas in treatments with peptidoglycan (PGN), the three response groups were more distantly related. In liver, TF and D were clustered in PBS-treated groups, whereas both Poly I:C and PGN treated groups both showed more divergence (Fig. 1b).

### Overview of differentially expressed genes

**Fig. 2:**
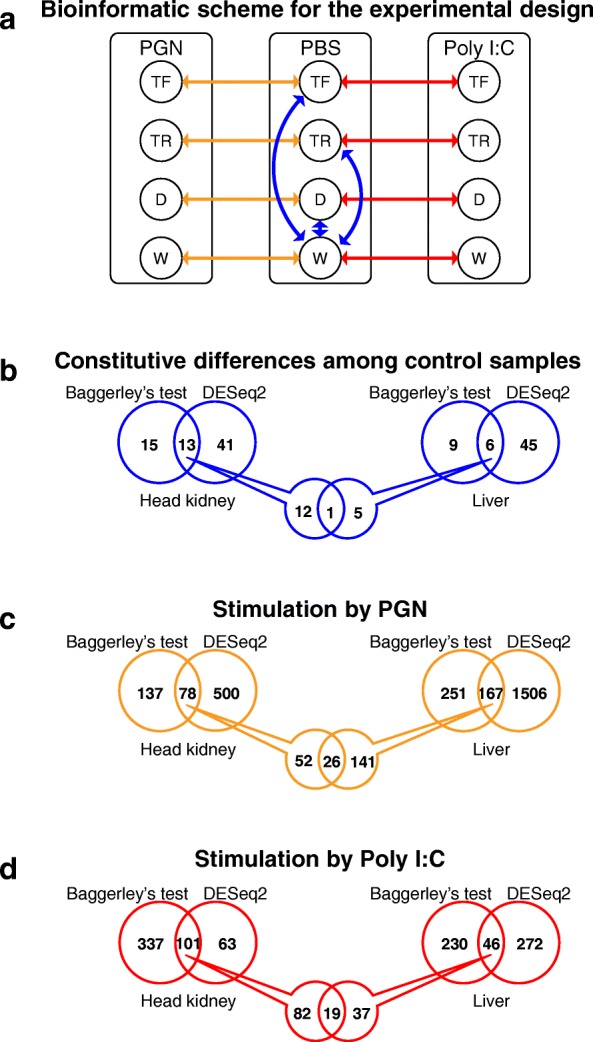
Bioinformatic analysis plan for the study. **a**) Pairwise assessment within each group, comparing immune-stimulated fish to their respective group treated with PBS, peptidoglycan (PGN) and Poly I:C. **b**) number of differentially expressed genes (DEGs) identified in the study by two different statistical normalized methods (Baggerley’s and DESeq2) treated with PBS, **c**0 peptidoglycan (PGN), and **d**) Poly I:C. Numbers refer to DEGs displaying a fold-change ≥3 among fish groups, with a normalized false discovery rate (FDR) *P*-value correction < 0.01). See Fig. 1 legend for abbreviations

We used two RNA-Seq normalization methods to establish significantly differentially expressed genes (DEGs) in this study, DESeq2 and Baggerley’s test (see Methods). To establish constitutive differences among the growth response groups in relation to the wild-type, we performed pairwise comparisons of control (i.e. PBS-injected) samples for i) D vs. W, ii) TF vs. W and iii) TR vs. W (Fig. 2b), revealing 129 DEGs for head kidney and liver, with 18 commonly identified by both normalization methods (Fig. 2b, Table 1). We also considered the effects of immune stimulation separately for each growth response group and tissue by comparing i) PGN vs. control and ii) Poly I:C vs. control for W, D, TF, and TR in liver and head kidney. A total of 3688 immune-responsive DEGs were detected, with 357 common to both approaches (Fig. 2c and d, Additional file 1: Table S1) that were used for further analysis. These results are expanded below.

**Table 1:** Differentially expressed genes (DEGs) among PBS injected TF, TR, and D groups relative to W. DEGs with a fold change ≥3 are shown in bold (significant by Baggerley’s test, normalized FDR correction *P*-value < 0.01)

Tissue	Gene	Annotation	Fold change		
			TR/W	TF/W	D/W
Head kidney	unigene22417332	Aquaporin 1	−1.5	1.1	**−3.5**
	unigene22417191	ATP-dependent RNA helicase DHX30-like	**6.4**	**3.1**	1.7
	unigene22417189	ATP-dependent RNA helicase DHX30-like	**4.9**	2.6	1.6
	unigene22386458	B chain crystal structure of avidin	2.8	**4.1**	−2.0
	unigene22392248	Complement C1q-like protein 4 precursor	**−3.6**	−1.9	−2.0
	unigene22390619	Leucine-rich repeat-containing protein 19	**4.6**	3.0	1.7
	unigene22396071	Methyltransferase DDB-like	**6.2**	**4.1**	**3.1**
	unigene22408729	Perforin-1-like isoform X1	−1.5	−1.1	**−3.7**
	unigene22418289	Sodium channel protein type 4 subunit alpha B	**53.9**	**11.9**	−2.4
	unigene22385893	Uncharacterized protein LOC109903204	−2.2	−1.4	**−5.2**
	unigene22389921	Uncharacterized protein LOC109904151	−1.5	−1.0	**−3.6**
	unigene22393685	Uromodulin	−1.3	1.1	**−3.6**
	unigene22425942	ORF2 protein	**3.8**	1.2	1.2
Liver	unigene22426268	Fatty acid-binding protein 1	−1.1	**−4.0**	−2.7
	unigene22387365	Microfibril-associated glycoprotein 4-like	**−3.2**	−1.5	1.3
	unigene22426266	Saxitoxin and tetrodotoxin-binding protein 1	**−3.5**	−1.5	1.3
	unigene22418289	Sodium channel protein type 4 subunit alpha B	**30.2**	**8.9**	−1.0
	unigene22391480	Transmembrane protein 116	**−8.9**	−2.3	1.0
	unigene22426238	C-C motif chemokine 19-like	**−4.3**	3.0	1.8

*PBS* phosphate-buffered saline, *PGN* peptidoglycan, *Poly I:C* polyinosinic-polycytidylic acid. W, non-transgenic (wild-type) coho salmon on a full satiation ration, *TF* GH transgenic coho salmon on a full satiation ration, *TR* GH transgenic coho salmon on restricted ration equal to that consumed by W, *D* domesticated coho salmon on a full satiation ration

### Constitutive differences in expression among growth-response groups

**Fig. 3:**
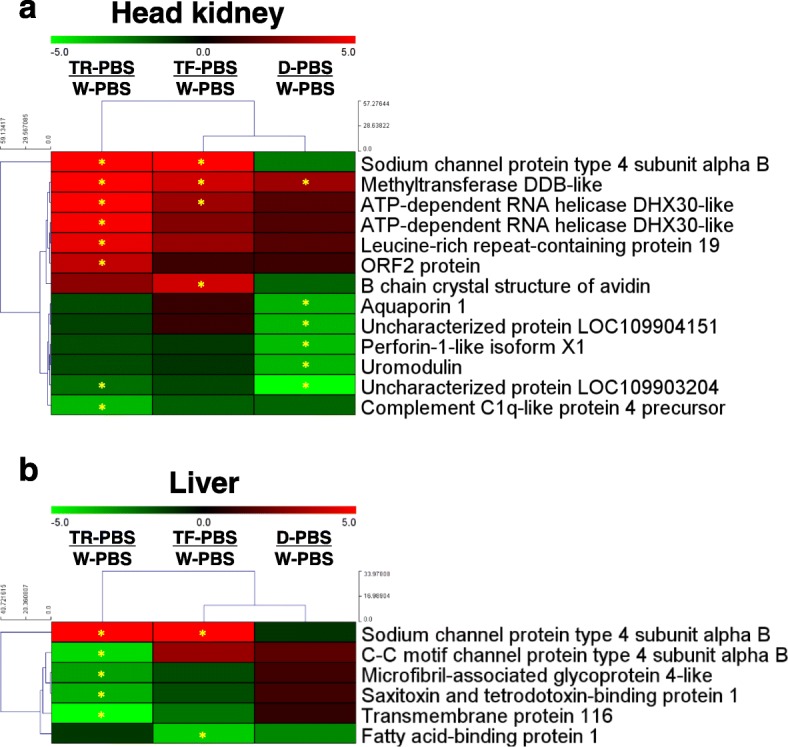
Heatmap of differentially expressed genes (DEGs) from comparisons among PBS-treated groups **a**) head kidney and **b**) liver. A star within cells refers to DEGs determined by the criteria of fold-change ≥3, and a normalized false discovery rate (FDR) *P*-value correction < 0.01. See Fig. 1 legend for abbreviations

Constitutive DEGs among control samples for TF, TR, and D relative to W were determined for head kidney and liver (Fig. 3; gene lists and fold change values shown in Table 1). In a cluster analysis of head kidney DEGs, TF and D clustered together to the exclusion of TR (Fig. 3a). There were 13 DEGs comparing W with the PBS-treated control groups for this tissue (Fig. 3a) with 11 annotated in the published coho salmon transcriptome [22]. Methyltransferase DDB-like was highly upregulated in all three groups relative to W (Fig. 3a; Table 1). The sodium channel protein type 4 subunit alpha B gene was very highly upregulated in both T groups (53.9/11.9-fold in TF/TR vs. W), whereas D showed reduced expression vs. W (Fig. 3a; Table 1). In TR, the genes encoding Leucine-rich repeat-containing protein 19, one ATP-dependent RNA helicase DHX30-like, and ORF2 protein were upregulated vs. W, while the gene encoding B chain crystal structure of avidin was more highly expressed (4.1 fold) in TF compared to W (Fig. 3a; Table 1). The gene encoding complement C1q-like protein 4 precursor had significantly lower expression in TR than W (Fig. 3a; Table 1). The genes encoding Aquaporin 1, Perforin-1-like and Uromodulin, and two additional uncharacterized protein products were significantly less transcriptionally abundant in D compared to W (Fig. 3a and Table 1).

As seen in head kidney, TF and D clustered together with respect to shared DEGs vs. W in liver (Fig. 3b). Moreover, 6 DEGs were identified with constitutive expression differences compared to W; 5 DEGs for TR, 2 DEGs for TF, and none for D (Fig. 3b). The sodium channel protein type 4 subunit alpha B gene, as in head kidney, showed highly upregulated expression in both TF (8.9-fold) and TR (30.2-fold) compared to W (Table 1). The genes encoding Saxitoxin and tetrodotoxin-binding protein, Microfibril-associated glycoprotein 4-like, C-C motif channel protein type 4 subunit alpha B, and Transmembrane protein 116, all had significantly lower reduced expression in TR vs. W, whereas fatty acid-binding protein 1 gene had lower expression in TF specifically (Fig. 3b).

### Growth response group responses to immune stimulation

**Fig. 4:**
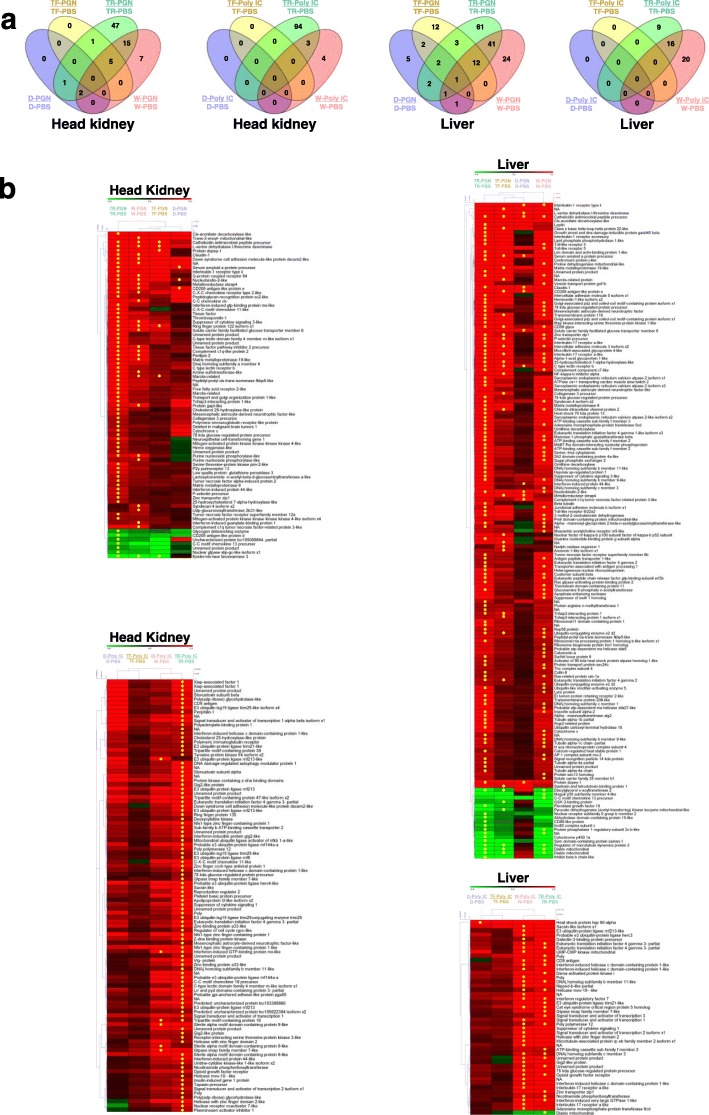
**a**) Number of differentially expressed gene (DEG) shared among comparisons within the fish groups (TF, TR, W, D) treated with immune stimulants Poly I:C, relative to each respective fish group treated with PBS, for both head kidney and liver. **b**) Heatmap for all significant differentially expressed gene (DEG) for comparison within fish groups treated with immune stimulants compared the same group treated with PBS for both head kidney and liver. Hierarchical clustering analysis was performed by MeV (ver. 4.9; https://sourceforge.net/projects/mev-tm4/files/mev-tm4/). A star within cells refers to DEGs determined by the criteria of fold-change ≥3, and a normalized false discovery rate (FDR) *P*-value correction < 0.01. See Fig. 1 legend for abbreviations

The number of DEGs arising from immunological stimulation (i.e. PGN or Poly I:C) relative to PBS-treated controls within each growth response group and tissue are shown in Fig. 4a and Table 2. A total of 391 DEGs were detected, with more DEGs (311) in head kidney than in liver (213 DEGs), and more DEGs for PGN (358) than Poly I:C (166). TR (316 DEGs) and W (152 DEGs) displayed many more immune responsive genes than TF (38 DEGs) and D (18 DEGs). A total of 57 genes showed responses in multiple treatments and tissues (Additional file 1: Table S1).

**Table 2:** Number of differentially expressed gene (DEG) determined from comparison of treated groups relative to their respective PBS-treated group. Treatments were the bacterial mimic peptidoglycan (PGN) and viral mimic Poly I:C. An arrow refers to up or down expression of DEG. A value in parenthesis is an average value of DEGs

Tissue	Comparison	D		TR		TF		W	
Head kidney	PGN-treated vs. PBS-treated	3	↑ 3 (6.6)	71	↑ 64 (11.0)	6	↑ 5 (17.3)	29	↑ 28 (9.7)
			–		↓ 7 (−3.8)		↓ 1 (−5.9)		↓ 1 (−5.5)
	Poly I:C-treated vs. PBS-treated	0	–	97	↑ 97 (6.6)	0	–	7	↑ 7 (7.1)
			–		–		–		–
Liver	PGN-treated vs. PBS-treated	14	↑ 14 (18.6)	123	↑ 109 (11.0)	32	↑ 27 (14.4)	80	↑ 72 (9.8)
			–		↓ 14 (−6.7)		↓ 5 (−4.8)		↓ 8 (−11.8)
	Poly I:C-treated vs. PBS-treated	1	↑ 1 (7.3)	25	↑ 24 (9.8)	0	–	36	↑ 36 (7.6)
			–		↓ 1 (−3.8)		–		–

*PBS* phosphate-buffered saline, *PGN* peptidoglycan, *Poly I:C* polyinosinic-polycytidylic acid. W, non-transgenic (wild-type) coho salmon on a full satiation ration, *TF* GH transgenic coho salmon on a full satiation ration, *TR* GH transgenic coho salmon on restricted ration equal to that consumed by W, *D* domesticated coho salmon on a full satiation ration

In head kidney and liver sampled after PGN treatment, TR showed the most DEGs (71/123), followed by W (36/116), TF (6/32) and D (3/14) (Table 2). The majority of genes were up-regulated in response to PGN, but there was extensive variation in responses among the different groups (Additional file 1: Table S1). Indeed, only one gene (cathelicidin antimicrobial peptide precursor) was commonly up-regulated in all fish groups in response to either immune mimic and either tissue (liver, PGN treatment; Additional file 1: Table S1). The gene encoding epidermis-type lipoxygenase 3 downregulated by PGN in head kidney in three growth response groups (TR, TF, and W, while three genes (encoding diacylglycerol O-acyltransferase 2 gene, regulator of microtubule dynamics protein 2 gene, and the diablo mitochondrial gene) showed downregulation in response to PGN in liver in TR, TF, and W (Additional file 1: Table S1).

For Poly I:C treated groups, few DEGs were detected in D (0 in head kidney and 1 in liver) and none in either tissue for TF. In contrast, TR showed many up-regulated genes (97 in head kidney and 25 in liver) and W had 7 in head kidney and 36 in liver). No downregulated DEGs were detected (Table 2 and Additional file 1: Table S1).

These data reveal a strong difference in response to Poly I:C between faster growing genotypes (TF and D) vs. slower growing groups (W and TR). Overall, the two strains with accelerated growth (TF and D, relative to W) each showed a reduced transcriptomic response to both immune mimics in liver and head kidney. Conversely, TR showed evidence for an augmented transcriptomic response to both immune mimics in liver and head kidney compared to W.

Cluster analysis showed that, among all DEGs, D and TF were grouped most closely for all tissues and treatments with the exception of head kidney treated with PGN where W and TF were clustered closely in the head kidney treated with PGN (Fig. 4b).

### Immunological pathway analysis using KEGG analysis

**Fig. 5:**
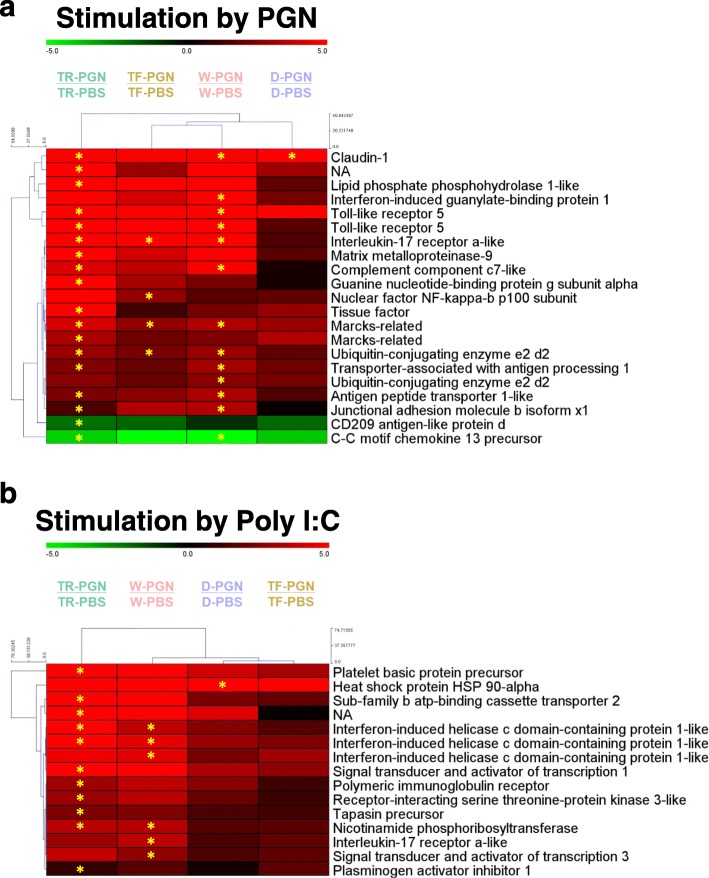
Heatmap for immune-related KEGG pathway-annotated differentially expressed gene (DEG) for both **a**) head kidney and **b**) liver for each comparison within the fish groups treated with PGN and Poly I:C. Hierarchical clustering analysis was performed by MeV (ver. 4.9; https://sourceforge.net/projects/mev-tm4/files/mev-tm4/). A star within cells refers to DEGs determined by the criteria of fold-change ≥3, and a normalized false discovery rate (FDR) *P*-value correction < 0.01. See Fig. 1 legend for abbreviations

Within the coho salmon transcriptome [22], KEGG analysis revealed that approximately 24,772 consensus sequences were significantly associated with KEGG ID codes. Among those, 12,294 sequences were matched to 7223 KEGG IDs for known metabolic or signalling pathways. Among those, 753 sequences were classified as immune-related amongst 16 immune pathways (data not shown). For comparisons between immune stimulated and PBS-treated fish within each group, 193 sequences were found as immune-related DEGs (Table 3). Among these 193 DEGs, 21 immune-related DEGs (9 in head kidney, 15 in liver, 3 in common) were found from PGN treatment (Table 3). For Poly I:C treatment, 15 DEGs (11 in head kidney, 8 in liver, 4 in common) were found as immune-related genes (Table 3). In cluster analyses, W and TF were closely related in the PGN treatment (Fig. 5a), whereas D and TF were clustered together for the Poly I:C treatment (Fig. 5b).

**Table 3:** Number of differentially expressed gene (DEG) associated with immune-related KEGG pathways determined in both head kidney and liver for peptidoglycan (PGN) and poly I:C treated fish, assessed by comparison of against PBS-treated fish in each same group. Total number of DEGs of each genotype were different from the sum of values because some genes have multi-functional characteristics by KEGG pathway analysis

Immune-related KEGG pathway		PGN-treated / PBS-treated									Poly I:C-treated / PBS-treated								
Full name	Abbreviation	Head kidney				Liver				TotalDEG	Head kidney				Liver				Total DEG
		D	TR	TF	W	D	TR	TF	W		D	TR	TF	W	D	TR	TF	W	
Antigen processing and presentation	APP						2		2	2		2			1				3
Complement and coagulation cascades	CCC		1				1		1	2		1							1
Cytosolic DNA-sensing pathway	CDs									0		1							1
C-type lectin receptor signaling pathway	CLRs		1					1		2		1				1			1
Chemokine signaling pathway	Cs		2						1	2		3				1		1	4
Fc gamma R-mediated phagocytosis	FGRP		2	1	1		1			3									0
Intestinal immune network for IgA production	IINIP									0		1							1
IL-17 signaling pathway	ILs		1				2	1	1	2					1			1	2
Leukocyte trans-endothelial migration	LTM		2		1	1	3		1	3									0
NOD-like receptor signaling pathway	NRs				1					1		3			1	2		1	4
Platelet activation	PA						1			1									0
RIG-I-like receptor signaling pathway	RRs									0		2				1		3	3
Th17 cell differentiation	TCD									0		1			1	1		1	3
Toll and Imd signaling pathway	TIs						1	1	2	2									0
Toll-like receptor signaling pathway	TRs						2		2	2		2				1			2
Th1 and Th2 cell differentiation	TTCD									0		1				1			1
Total			9	1	3	1	13	3	10	21		18			4	8		7	15
Average fold change			3.0	3.3	6.9	10.9	15.8	5.5	2.8	9.0		12.8			7.3	7.9		4.2	16.0

*PBS* phosphate-buffered saline, *PGN* peptidoglycan, *Poly I:C* polyinosinic-polycytidylic acid. W, non-transgenic (wild-type) coho salmon on a full satiation ration, *TF* GH transgenic coho salmon on a full satiation ration, *TR* GH transgenic coho salmon on restricted ration equal to that consumed by W, *D* domesticated coho salmon on a full satiation ration

## Discussion

The present study has examined the transcriptomic response to immune stimulation in four coho salmon groups possessing different growth rates arising from domestication, GH transgenesis and ration level. Transcriptome-level alterations caused by treatment with pathogen mimics were found to be very complex, affecting multiple pathways to various degrees, similar to results seen in other studies [23, 24]. Immune challenges with bacterial and viral mimics previously have been used to explore tissue and time-dependent responses to identify tissue-specific immune processes involved in different activation phases of an infection [25–28]. Treatment conditions (viral vs. bacterial mimics) and fish group (growth response group and environmental influences) were all found to affect the pathways in distinct ways, including many responses that depended on interacting factors.

### Basal level gene expression differences

To determine whether the four fish growth response groups (W, TF, TR, D) had any innate basal differences in gene expression, we first compared PBS-treated groups lacking immune stimulant treatment. Among 18 DEGs identified, perforin-1-like isoform X1 in head kidney and C-C motif chemokine 19-like gene in liver were identified as immune-related genes. Perforin-1, which is involved in natural killer cell mediated cytotoxicity, was significantly down-regulated in expression in D only (− 3.7-fold change relative to W). In mammals, perforin plays a central role in secretory granule-dependent cell death induced by natural killer T cells and cytotoxic T lymphocytes, important for defence against virus-infected or neoplastic cells [29, 30]. Perforin also has a structural similarity with the ninth component of complement (C9) [31], and plays an important role in killing cells that are recognized as non-self by the immune system [32]. For fish perforin genes, limited studies have been reported. In zebrafish, 6 perforin genes were characterized [33] with potential for multiple functions. In Olive flounder (*Paralichthys olivaceus*), a significant increase of perforin expression in head kidney was observed in the early developmental stage, suggesting that perforin may play a key role in the innate larval immune system [34]. Flounder and carp (*Cyprinus carpio*) show specific cell mediated cytotoxicity using mainly a perforin/granzyme-like pathway [35, 36]. The head kidney is an important organ with key regulatory functions and is a central organ for immune-endocrine interactions and neuroimmunoendocrine connections [37, 38]. It is unclear whether down-regulation of the perforin-1 like gene in D fish observed in the present study leads to an attenuated innate immune response. However, that perforins play a crucial role in immune signalling pathways suggests that further functional studies on this gene would be of value to elucidate the molecular regulatory mechanisms of its action in response to strains with different growth rates that may cause trade-offs with immune function. The differential response of strains examined here with respect to perforin expression suggests that mechanisms affecting immune function arise by separate mechanisms, at least in part, during transgenesis, domestication, and ration restriction.

The C-C motif chemokine 19-like gene in liver showed a significant decrease in expression in TR fish compared to other groups. This gene plays a role in the chemokine signalling pathway, but has only been studied in a few fish species such as turbot (*Scophthalmus maximus*), striped murrel (*Channa striatus*), channel catfish (*Ictalurus punctatus*), and ayu (*Plecoglossus altivelis*) [39–42]. In these studies, the C-C motif chemokine 19-like mRNA expression was highly upregulated upon bacterial and viral infection, consistent with findings in the present study using bacterial and viral mimics. Distinct from the overall elevation of immune response seen in TR, the significantly lower basal level expression of the C-C motif chemokine 19-like gene in TR fish may arise from nutritional insufficiency and energy imbalance that prevents full development of this immunological response by reducing the ability to mobilize immune cells to a site of infection. While further investigation is required, this observation suggests that in some cases GH overexpression in the absence of adequate nutritional input may cause pathological response to immune stimulation as is observed in other species and strains that possess balanced energy status.

The high expression of the sodium channel protein type 4 subunit alpha B gene in both tissues in TF and TR groups is intriguing. In general, sodium channel protein type 4 subunit alpha protein is expressed in skeletal muscle, neurons, and other tissues, and is known to play a role in the generation and propagation of action potentials in neurons and in muscle in animals. It provides a critical function, and mutation of this gene leads to several myotonia and periodic paralysis disorders [43–45]. Previous research has found that GH over-expression can have very broad pleiotropic effects on many pathways related to physiological, morphological, and behavioural phenotypes of the organism [20, 25]. The strong response of sodium channel protein type 4 subunit alpha B gene to GH transgenesis may be another example of pleiotropic responses, or this gene may be involved in some yet unknown immune response process.

### Metabolic and growth gene pathway differences

Differences in metabolic and growth gene pathways among the groups prior to treatment were also examined to identify differences that may result in trade-offs with immune function (i.e. growth vs. immunity) [7, 11, 46]. In the present study, two ATP-dependent RNA helicase DHX30-like genes in head kidney were identified to have a molecular function related to metabolism. RNA helicases generally act as components of multi-protein complex with additional ATP-independent roles presumably conferred through their interactions with protein partners [47], while also playing an important role in the assembly of the mitochondrial large ribosomal subunit [48]. In the present study, significantly higher expression of two ATP-dependent RNA helicase DHX30-like genes was seen in TF and TR vs. W when compared to D vs. W, suggesting the high level of GH produced in both GH transgenic salmon groups [17] may be influencing this pathway. In human cells, overexpression of ATP-dependent RNA helicase DHX30-like gene causes high production of viral Gag proteins and elevates the production of virus particles, leading to enhanced human immunodeficiency virus type 1 transcription [49]. It is not clear if overexpression of basal ATP-dependent RNA helicase DHX30-like mRNA would affect T (GH transgenic) salmon immunodeficiency, but further studies on this gene would be of value to assess trade-offs in a range of environmental conditions.

Previous studies comparing GH transgenic and non-transgenic fish have found significant effects on lipid metabolism pathways [11, 46, 50, 51]. In the present study, fatty-acid binding protein 1 was significantly down-regulated in TF liver compared to other groups. Fatty-acids affect many aspects of cellular function as an energy source and as signals for metabolic regulation, modulation of gene expression, growth and survival pathways, and inflammatory and metabolic responses [52, 53]. The fatty-acid binding protein 1 is known as a liver-fatty-acid binding protein, and its mRNA level is increased by fatty acids, dicarboxylic acids and retinoic acid. Effects relating to fatty-acid metabolism have also been reported in other GH transgenic salmonids using the OnMTGH1 transgene [13]. For both amago salmon (*Oncorhynchus masou*) and Arctic charr (*Salvelinus alpinus* L.), reduced D6-desaturase has been observed, an enzyme involved the innate immune systems [54, 55]. These data may be species-specific as reduced expression of D6-desaturase was not seen in present study. Using microarray analysis, enhanced expression of genes in hepatic tissues have also been seen in GH transgenic amago salmon, specifically NADH dehydrogenase, leucite-derived chemotaxin2, and complement factor H [54]. These genes were stimulated in TF in liver in the present study (data not shown, > 2-fold change). In case of lectin, this gene showed strongly reduced expression in GH transgenic amago salmon [54], but increased expression in TF coho salmon in the present study, again indicating species-specific responses. In a previous study with salmon (*Salmo salar*), lectin was strongly up-regulated during infection [56], corresponding with a previous result with GH transgenic coho salmon [19] and with the present study.

### Immune response differences upon immunological stimulation

Previous studies examining domesticated and transgenic coho salmon using microarray technology have found that gene expression profiles, relative to wild type, were highly correlated and revealed changes in multiple processes including e.g. energy metabolism of carbohydrates and lipids, cellular structure, and immune function [11, 57]. Domesticated salmon have been shown to have elevated GH and IGF-I (insulin-like growth factor) levels relative to wild type [7, 10, 11], albeit not as high as seen in GH transgenic animals [17]. Thus, many changes in these strains have been found to affect pathways similarly. Consistent with previous microarray studies, correlation analysis indicated that the overall pattern of gene expression in GH transgenesis and domestication, relative to wild strain, were affected to a significant extent in parallel ways. Interestingly, the fully-fed transgenic group (TF) showed higher correlations with the domesticated strain (D) than did ration-restricted transgenic salmon group (TR), indicating that nutritional status also may significantly affect homeostasis of energy balance and gene regulation in transgenic salmon. Further, although both D and TF strains both possess enhanced growth relative to wild type, some differences were observed between the strains suggesting that effects on immune pathways can be strain-specific. This would be expected because domestication arises from the gradual selection of variation within a strain over multiple generation with the opportunity for co-selection of other traits that can compensate for negative pleiotropic effects. In contrast, GH transgenesis is a powerful and immediate influence on the physiology of an animal whose genome has evolved for lower growth rates, and thus such animals are likely to experience more pleiotropic effects and have limited capacity to compensate for negative epistatic interactions caused by suddenly modified growth and metabolic pathways.

In several previous studies, TR coho salmon have shown uncoupling of GH and amino acid metabolism signals caused by long-term nutritional insufficiency affecting expression of genes associated with multiple pathways [18, 58–60]. These additional costs and trade-offs have the potential to cause TF and TR salmon to encounter critical energy imbalance which in turn could reduce energetic support of essential immunological mechanisms needed to cope with infection.

**Fig. 6:**
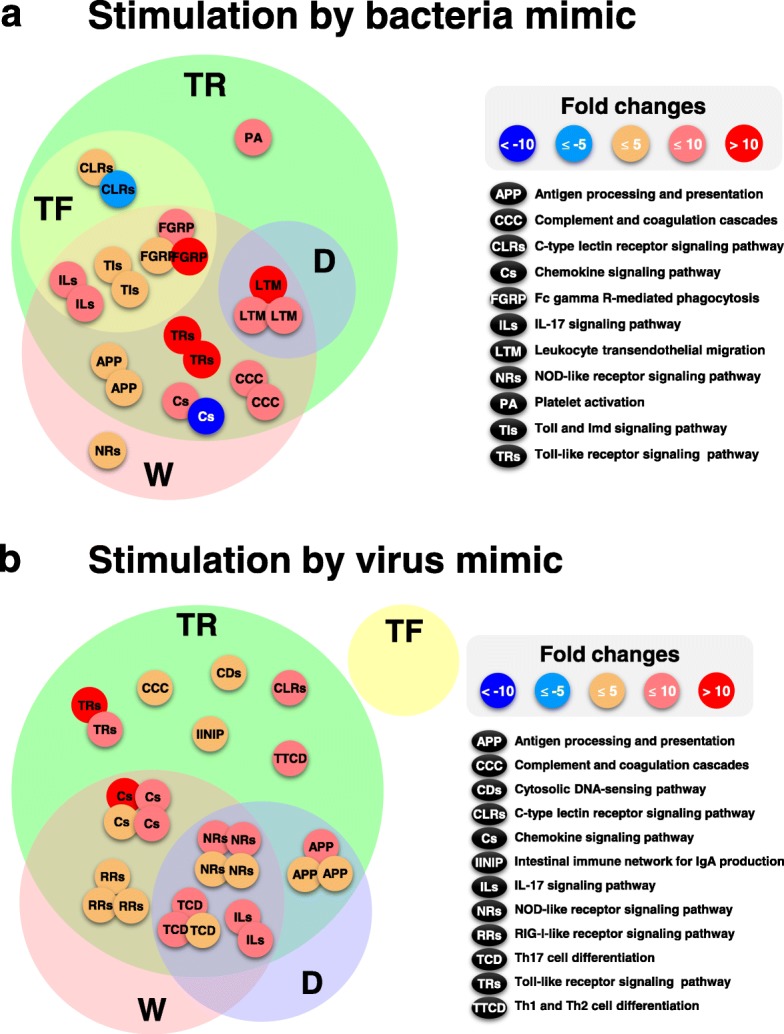
Diagram illustrating relationships of significant immune-related KEGG pathway differentially expressed gene (DEG) identified within the fish groups treated with **a**) bacterial and **b**) viral mimics. The number and size of circles within the figure corresponds to the number of DEG in the affected pathway. Overlapping circles represent shared responses. The color of each circle refers to the value of gene expression according to the fold change. See Fig. 1 legend for abbreviations

An overall similar response of immune-relevant genes between T and D has been observed, but pathway-specific differences were also found (e.g. chemokine signalling pathway), suggesting the presence of non-parallel responses to immune stimulation among strains (Fig. 6). TF has fewer immune responses than TR and D, and in particular, there were no significant immune-related DEGs in TF in liver for both pathogen mimics, indicating disease resistance of these animals in culture is likely reduced (at least relative to W [19]) and they may be less able to respond to pathogen exposure. A greater number of immune-relevant genes were up-regulated in TR than in TF, suggesting that TR may have enhanced capacity to respond to infection to a greater extent than seen for TF. TF salmon possess an elevated basal metabolic rate [61] relative to wild salmon and unsatiated T salmon, and this overall enhancement of metabolism may affect energy available for immune responses. TF salmon have been shown to have reduced disease resistance [19, 20] and it is possible this arises from hyper-fast growth preventing development of a full immune response, whereas in TR animals where growth is maintained at a lower wild-type rate by ration restriction, a higher immune response may be physiologically possible. Indeed, it is well-known that a rapid growth rate and immunity showed an inverse relation in aquaculture [62–64]. Previous studies examining immune and growth-related gene expression in muscle of W, TF, TR and D groups treated with Poly I:C or PGN found complex responses depending on the pathways examined [21]. Strikingly, PGN treatment induced a strong pro-inflammatory response [e.g. TNF-α (tumor necrosis factor-alpha) among others] in all groups but TF, and that TF salmon had higher basal levels of expression suggesting this latter strain may be experiencing a chronic inflammatory response and possesses little ability for further stimulation. Poly I:C treatment was found to induce viral-response genes in all groups but TF, again suggesting a dampened response in this fast-growing salmon group as seen in the present RNA-Seq study in head kidney and liver. This study also noted that PGN and Poly I:C modified the expression GH axis genes which, coupled with the effects of GH overexpression seen in the present study, suggests significant cross-talk exists between growth and the immune system. Consistent with this conclusion, enhancement of energy-sensing AMPK (AMP-activated protein kinase) subunits has been observed in fast growing transgenic salmon [65], and immune stimulation was seen to reduce expression of several AMPK subunit-encoding genes specifically in GH-transgenic fish, confirming the interaction between growth and immune pathways.

The data in the present work extend previous findings that showed the innate immune system of this GH transgenic strain (TF) was less effective (in response to *Aeromonas salmonicida* treatment), whereas the acquired immune response could provide full immunity [20]. Further, higher susceptibility of GH transgenic coho salmon to a bacterial (*Vibrio anguillarum*) challenge has been observed relative to that seen in wild-type [19]. In GH transgenic carp, elevated lysozyme and serum bactericidal activity have been observed, suggesting that disease resistance may be enhanced [66], whereas treatment with GH protein has shown complex modulations of immune responses in several fish species [67–71]. Together, species- and strain-specific immunological responses have been detected in fishes exposed to elevated GH.

Analyses of immune-related DEGs within groups of coho salmon demonstrating different growth responses found that most pathways were up-regulated in response to bacterial and viral mimic stimulations with the exceptions of c-type lectin receptor signalling (CLRs) and chemokine signalling pathways (Fig. 6). In particular, the CLRs from bacterial stimulation responded only in transgenic fish groups (TF and TR), suggesting that the CLRs pathway would be a valuable direction for further research to understand how GH influences shaping the immune response to pathogens in growth modified animals. The effects in NOD-like receptor signalling pathway (NRs) in W, platelet activation in TR, and no immune responses in TF, show group specific responses to immune simulants, and as such these responses provide avenues to begin to further dissect differences between GH transgenic and domesticated strains.

## Conclusion

This study has examined trade-offs between growth rate and immune function arising from anthropogenic enhancement of growth rates by selective breeding and genetic engineering (GH transgenesis) and has found significant interaction between these two critical pathways. The findings are multi-faceted and improve our understanding of the pleiotropic effects of growth modification on the immunological responses in fish, revealing that different genetic modification approaches and rearing conditions (i.e. nutritional state) influence gene expression profiles and pathways in unique complex ways (Fig. 6). The study also identified a strong positive response to ration restriction on immune function in the GH transgenic salmon group which warrants further study in other growth accelerated strains. These data will assist with development of strains and culture conditions for aquaculture by allowing development of genetic markers that reveal effects on immune function in response to programs seeking to enhance growth rate. In addition, the information identified in this study improve our understanding of effects of growth on immune function and thereby fitness to aid in ecological risk assessments of modified strains (transgenic, domesticated, or other) that have the potential to introgress into natural populations.

## Methods

### Experimental set up

Experiments were performed at Fisheries and Oceans Canada (Department of Fisheries and Oceans; DFO) in West Vancouver, Canada. This facility is designed with containment measures to prevent the escape of genetically-modified fish to the natural environment. All experiments were performed in compliance with the Canadian Council on Animal Care guidelines under a permit from DFO’s Pacific Regional Animal Committee (Animal Use Permit 12–017). Four size-matched populations of juvenile coho salmon, *Oncorhynchus kisutch,* were generated: (i) 19-month-old wild-type coho salmon fed to satiation (W), (ii) 10-month-old non-transgenic domesticated coho salmon selected for rapid growth and fed to satiation (D), (iii) 6-month-old GH transgenic coho salmon fed to satiation throughout life and possessing elevated growth rate relative to W (TF) [72], and (iv) 17-month-old GH transgenic salmon that were ration-restricted to the W satiety level, leading to wild-type growth rate throughout life (TR). TR fish were produced by pair feeding them (at each feeding session) the same amount of food that had immediately prior been consumed by the W group that had been fed to satiation. Satiation was defined as the condition when three singly offered food pellets reached the bottom of the tank without consumption. Under these conditions, and because of the vigorous appetite of transgenic fish, this resulted in consumption of the same amount of food by the W and TR groups. Using fish of different ages was necessary to standardize confounding effects of body size on gene expression, due to the highly different growth rates among groups. All groups of fish, *n* = 500 individuals (125 fish per each group) were maintained under the same standard conditions (4000 L tanks supplied with 10.5 ± 1 °C aerated well water, natural photoperiod, fish density less than 5 kg/m^3^) and were fed commercial salmonid diets (Skretting Canada Ltd.) twice daily at 9:00 a.m. and 15:00 p.m. For each size matched group, *n* = 60 individuals (W: 74.2 ± 3.6 g, D: 77.9 ± 0.5 g, TF: 77.9 ± 6.1 g, TR: 78.6 ± 3.3 g) were distributed into four separate 70 L tanks. Within each tank, three experimental groups were uniquely marked and then intraperitoneally injected with the following treatments: i) *n* = 24 per tank with polyinosinic-polycytidylic acid (Poly I:C) at 200 μg per 100 g fish weight, ii) n = 24 per tank with peptidoglycan (PGN) at 200 μg per 100 g fish weight and iii) n = 24 per tank with phosphate-buffered saline (PBS) as a control. After treatment, all fish were re-stocked back into 4000 L tanks and maintained under the common garden design described above. The concentrations of Poly I:C and PGN used were based on past work [73–77].

### Sampling and RNA extraction

For each growth response group (TF, TR, W, and D), 10 fish were sampled 6 h and 30 h post-treatment. Individual fish were rapidly euthanized with a lethal concentration of tricaine methanesulfonate (200 mg/L; Syndel Laboratories Ltd., Vancouver, BC, Canada; buffered in 400 mg/L sodium bicarbonate) after initial sedation using Aquacalm (1 mg/L; Syndel Laboratories Ltd., Vancouver, BC, Canada). A range of different tissues, including head-kidney, intestine, liver, skeletal muscle, and spleen, were rapidly team dissected (< 3 min per fish) and stored in RNAlater™ (ThermoFisher Scientific) overnight at 4 °C, followed by long-term storage at − 20 °C. For this study, total RNA was extracted from head kidney and liver samples at the 30 h time point using RNeasy mini kits (Qiagen, Valencia, CA, USA). Concentration and purity of the RNA for each sample was measured using a Nanodrop (Thermo Scientific, Wilmington, DE, USA), and RNA integrity confirmed using an Agilent 2100 Bioanalyzer (Agilent Technologies, Palo Alto, CA, USA). Five individual RNA samples were randomly selected from each group per treatment for RNA-Seq analysis.

### RNA-Seq analysis

High-quality RNA (RNA integrity number > 9.0) preparations were quantified using an Invitrogen Qubit Fluorometer and Agilent 2100 Bioanalyzer. The sequencing libraries were made from 2 μg of pooled RNA (0.4 μg per fish from each of 5 fish per pool), creating two biological replicates per growth response group (i.e. *n* = 2 pools, each of *n* = 5 fish per treatment. Libraries (200 bp short-insert) were made with the TruSeq™ RNA sample preparation kit (Illumina, San Diego, CA, USA). Sequencing was conducted using the Illumina HiSeq2000 platform to generate 50-bp single-end reads by the Beijing Genomics Institute (BGI, Shenzhen, China). A total of 617,779,232 reads were generated. After removal of adaptor sequences, ambiguous nucleotides (*N* ≥ 10%), low-quality reads (where > 50% of bases had quality value scores ≤5) and sequences less than 15 bp, ~ 561 million reads (head kidney: 276,802,892, liver: 284,410,895) totalling 27.5 billion bases were obtained using the filter_fq software (BGI internal software) for further analysis.

For differential expression (DE) analysis, two different pipelines were used in this study. First, the RobiNA pipeline [78] was used, including for quality checks using default parameters. Within RobiNA, Bowtie 2 [79] was used for read mapping (mismatch cost = 2) against a reference coho salmon transcriptome [22]. Subsequently, DE analysis was performed in DESeq2, which assumes a negative binominal distribution of count data [80]. Second, CLC Genomics Workbench (Ver. 8.0.2) was used following a previous approach [81]; here, imported clean reads were mapped against the reference transcriptome, the insert size for paired-end reads was set between 150 and 250 bp and RPKM normalization of expression values was performed [81]. Identification of DE genes (DEGs) using the CLC approach was based on the RPKM values analysed using Baggerley’s test [82]. For both the DESeq2 and CLC approach, DEGs were filtered using a false-discovery correction rate (*P* < 0.01) and fold change cut-off ≥3. Pairwise comparisons made within growth response groups are shown in Fig. 2a. Principal component analysis was performed using CLC Genomics Workbench (Ver. 8.0.2). We note that the methods used here for normalization of RNA-Seq data present expression relative to the pool of sequenced transcripts in a given sample rather than as an absolute measure of gene expression per cell. DEGs were identified after comparing expression in treatment groups to normalized expression seen in wild type, and as such provide a relative measure of gene expression among treatment groups. We note that raw read numbers obtained for RNA-Seq were highly similar among groups (Additional file 2: Table S2).

For the following described analysis, commonly detected DEGs identified by both normalization methods were used. We chose to analyze only those genes found significant by both methods to focus our analysis on the most analytically robust DEGs. Using Blast2GO v3.1 [83], DEGs were assigned gene ontology (GO) terms for ‘biological process’, ‘cellular component’ and ‘molecular function’. The KAAS, Kyoto Encyclopedia of Genes and Genomes (KEGG) automatic annotation server [84, 85] was used for pathway analysis, focused on signalling and hormone pathways related to the immune response.

### Real-time quantitative PCR validation of RNA-Seq data

The same samples used in RNA-Seq (*n* = 2 pools of 5 fish per growth response group/treatment) were subjected to qPCR validation for a subset of DEGs. First-strand cDNA was synthesized from total RNA (0.5 μg) using the High Capacity cDNA synthesis kit with RNase inhibitor (Applied Biosystem, Foster City, CA, USA). Primers for qPCR (Additional file 3: Table S3) were designed with sequences from the coho salmon transcriptome [84] and checked for secondary structures using NetPrimer (http://www.premierbiosoft.com). All pairs of primers were validated for specificity by electrophoresis to confirm the expected amplicon size. Quantitative PCR (qPCR) was performed using 10 μl of Fast SYBR Green Master Mix (Invitrogen) with 0.2 μM/l of each primer, 5 μl of 20-fold diluted cDNA and nuclease-free water (Gibco, Carlsbad, CA) to a final volume of 20 μl in 96-well plates (Applied Biosystem, Forster City, CA). The reaction was performed in triplicate using the 75 Fast Real time PCR System (Applied Biosystem) with the reaction conditions: 95 °C/10 min; 40–45 cycles of 95 °C/2 s, 60 °C/15 s, 72 °C/33 s. After qPCR, a melt curve analysis was performed to verify the presence of a single amplicon peak. Levels of mRNA were calculated relative to the Ct value obtained for the reference gene (*Ubiquitin*) using the 2^-ΔΔCt^ method [86]. *Ubiquitin* was chosen for normalization as it possessed the most stable mRNA levels for the growth response and treatment groups among three potential reference genes examined [*β-actin*, *Ef-1a* and *Ubiquitin* (Additional file 3: Table S3)]. This normalization procedure accounts for differences in the proportion of mRNA relative to total RNA in a cell among groups.

### qPCR validation

qPCR analysis was performed for 8 early response and immune-associated genes (encoding Mx2 protein, Serum amyloid A-5 protein, Interleukin-8, Hepcidin, Radical S-adenosyl methionine domain-containing protein 2 precursor, Immune-responsive gene 1 protein homolog, TNF receptor superfamily member 5A, and MHC class I alpha chain) in order to supplement and validate RNA-Seq analysis (Additional file 4: Figure S1).

## Supplementary information

**Additional file 1: Table S1.** Differentially expressed gene (DEGs) list from head kidney and liver following immune stimulation (PGN or Poly I:C) of different growth response groups (D, TR, TF) of coho salmon relative to PBS-treated. The genes in bold refer to be found in both liver and kidney.

**Additional file 2: Table S2.** Raw reads of each group, tissue, and treatment.

**Additional file 3: Table S3.** Primers and probes used in this study.

**Additional file 4: Figure S1.** Relationship between mRNA expression level from qPCR analysis vs. normalized read number (RPKM) from RNA-Seq analysis, for 8 early responses and immune-associated genes (*Complement C*, *IL-8*, *IRG1*, *MHC 1a*, *Mx*, *Radical S*, *SAA*, *TNFR-5B*).

## Data Availability

The datasets generated and/or analysed during the current study are available in the NCBI repository with an accession number (BioProject, PRJNA595068).

## Abbreviations

AMPK: AMP-activated protein kinase
CLRs: C-type lectin receptor signaling pathway
D: Domesticated coho salmon on a full satiation ration
DEG: Differentially expressed gene
GH: Growth hormone
IGF: Insulin-like growth factor
NRs: NOD-like receptor signaling pathway
PBS: Phosphate-buffered saline
PCA: Principal component analyses
PGN: Peptidoglycan
Poly I:C: Polyinosinic-polycytidylic acid
T: GH transgenic coho salmon
TF: GH transgenic coho salmon on a full satiation ration
TNF: Tumor necrosis factor
TR: GH transgenic coho salmon on restricted ration equal to that consumed by W
W: Non-transgenic (wild-type) coho salmon on a full satiation ration
