# Supplementary material for: Effect of growth rate on transcriptomic responses to immune stimulation in wild-type, domesticated, and GH-transgenic coho salmon
Source: BMC Genomics. 2019 Dec 27;20:1024. doi: 10.1186/s12864-019-6408-4 (PMC6935076; doi:10.1186/s12864-019-6408-4)
Supplement: Supplementary file 1 — Additional file 1: Table S1. Differentially expressed gene (DEGs) list from head kidney and liver following immune stimulation (PGN or Poly I:C) of different growth response groups (D, TR, TF) of coho salmon relative to PBS-treated. The genes in bold refer to be found in both liver and kidney. [file 12864_2019_6408_MOESM1_ESM.docx]

Table S1. Differentially expressed gene (DEGs) list from head kidney and liver following immune stimulation (PGN or Poly I:C) of different growth response groups (D, TR, TF) of coho salmon relative to PBS-treated. The genes in bold refer to be found in both liver and kidney.

| Treatment | Tissue | Gene ID | Relative to PBS-treated | | | |  |
| --- | --- | --- | --- | --- | --- | --- | --- |
|  |  |  |  |  |  |  | Annotation |
|  |  |  | D | TR | TF | W |  |
| Peptidoglycan | Head kidney | **unigene22406570** |  | 11.1 |  | 8.3 | Nucleobindin-2-like |
| (PGN) |  | **unigene22404077** |  | 59.6 | 47.6 | 35.2 | Cathelicidin antimicrobial peptide precursor |
|  |  | unigene22398575 |  | 5.5 | 3.3 | 3.8 | Marcks-related |
|  |  | unigene22387268 |  | 8.1 | 5.2 | 6.6 | Ring finger protein 122 isoform x1 |
|  |  | **unigene22386313** |  | 40.4 | 18.6 | 37.2 | L-serine dehydratase l-threonine deaminase |
|  |  | unigene22402703 |  | -5.3 | -5.9 | -5.5 | Epidermis-type lipoxygenase 3 |
|  |  | **unigene22426055** |  | 42.8 | 12.0 |  | Protein dopey-1 |
|  |  | unigene22426915 |  | 25.0 |  | 17.1 | NA |
|  |  | **unigene22405218** | 8.9 | 8.6 |  | 5.2 | CD209 antigen-like protein e |
|  |  | unigene22424853 |  | 15.5 |  | 8.6 | G-protein coupled receptor 84 |
|  |  | **unigene22396321** |  | 28.6 |  | 7.4 | Down syndrome cell adhesion molecule-like protein dscam2-like |
|  |  | **unigene22400741** |  | 10.6 |  | 6.8 | Metalloreductase steap4 |
|  |  | unigene22418758 |  | -3.1 |  |  | PREDICTED: uncharacterized protein LOC105009844, partial |
|  |  | unigene22416726 |  | -3.8 |  |  | Nuclear GTPase slip-GC-like isoform x1 |
|  |  | **unigene22415017** |  | 8.2 |  | 6.2 | Suppressor of cytokine signaling 3-like |
|  |  | **unigene22391669** |  | 6.4 |  | 6.3 | Solute carrier family facilitated glucose transporter member 6 |
|  |  | **unigene22391652** |  | 5.3 |  | 5.1 | Matrix metalloproteinase-19-like |
|  |  | unigene22386096 |  | 5.5 |  | 3.7 | Amine sulfotransferase-like |
|  |  | **unigene22393641** |  | 33.4 |  | 13.3 | Claudin-1 |
|  |  | **unigene22397018** |  | 122.9 |  |  | Cis-aconitate decarboxylase-like |
|  |  | **unigene22397285** | 7.8 | 18.8 |  |  | Serum amyloid a protein precursor |
|  |  | **unigene22398493** |  | -3.8 |  |  | C-C motif chemokine 13 precursor |
|  |  | unigene22402400 |  | 5.9 |  | 4.8 | Tissue factor pathway inhibitor 2 precursor |
|  |  | unigene22418209 |  | 5.4 |  | 5.3 | Complement c1q-like protein 2 |
|  |  | unigene22411190 |  |  |  | 36.3 | Trans-2-enoyl- mitochondrial-like |
|  |  | unigene22404640 |  | 3.3 |  |  | Tumor necrosis factor receptor superfamily member 12a |
|  |  | unigene22415559 |  | 4.5 |  | 4.9 | Purine nucleoside phosphorylase-like |
|  |  | unigene22424064 |  | 8.1 |  |  | Thrombospondin-1 |
|  |  | **unigene22396514** |  | 14.6 |  |  | Interleukin-1 receptor type II |
|  |  | unigene22396077 |  | 8.0 |  | 3.9 | C-X-C chemokine receptor type 2-like |
|  |  | unigene22387411 |  | 4.6 |  | 5.1 | Protein gapt-like |
|  |  | unigene22396044 |  | -3.1 |  |  | Unnamed protein product |
|  |  | unigene22402460 |  | 6.0 |  | 5.3 | Perilipin 2 |
|  |  | unigene22407395 |  | 6.1 |  |  | Unnamed protein product |
|  |  | **unigene22410673** |  | 5.3 |  |  | Peptidyl-prolyl cis-trans isomerase fkbp5-like |
|  |  | unigene22422525 |  | -3.2 |  |  | Glycogen debranching enzyme |
|  |  | **unigene22405217** |  | 6.3 |  |  | C type lectin receptor b |
|  |  | unigene22415558 |  |  |  | 3.6 | Purine nucleoside phosphorylase-like |
|  |  | unigene22399830 |  | 9.9 |  |  | Tissue factor |
|  |  | **unigene22392798** |  | 6.8 |  |  | C-type lectin domain family 4 member m-like isoform x1 |
|  |  | unigene22388918 |  | 3.9 |  |  | Lactosylceramide -n-acetyl-beta-d-glucosaminyltransferase a-like |
|  |  | **unigene22400068** |  |  |  | 12.8 | Complement c1q tumor necrosis factor-related protein 3-like |
|  |  | unigene22396427 |  |  |  | 8.3 | CC chemokine ck- |
|  |  | unigene22387201 |  | 4.7 |  |  | Transport and golgi organization protein 1-like |
|  |  | unigene22396679 |  | 5.4 |  |  | Free fatty acid receptor 2-like |
|  |  | unigene22405435 |  | 4.4 |  |  | Low quality protein: glutathione peroxidase 3 |
|  |  | **unigene22422936** |  | 3.6 |  |  | Matrix metalloproteinase-9 |
|  |  | **unigene22404772** |  | 3.5 |  |  | 25-hydroxycholesterol 7-alpha-hydroxylase-like |
|  |  | unigene22409292 |  | 3.6 |  |  | Unnamed protein product |
|  |  | unigene22420508 |  | 4.6 |  |  | Neuroepithelial cell-transforming gene 1 |
|  |  | **unigene22416457** |  | 12.5 |  |  | Interferon-induced gtp-binding protein mx-like |
|  |  | unigene22416674 |  | 3.2 |  |  | Mitogen-activated protein kinase kinase kinase kinase 4-like isoform x4 |
|  |  | unigene22404560 |  |  |  | 3.5 | Interferon-induced guanylate-binding protein 1 |
|  |  | unigene22412094 |  | 3.8 |  |  | Tumor necrosis factor alpha-induced protein 2 |
|  |  | unigene22424894 |  | 4.0 |  |  | Cytochrome c |
|  |  | **unigene22426238** |  | 5.2 |  |  | NA |
|  |  | unigene22412374 |  | -4.4 |  |  | CD209 antigen-like protein d |
|  |  | **unigene22395558** |  | 4.3 |  |  | Mesencephalic astrocyte-derived neurotrophic factor-like |
|  |  | unigene22387496 |  | 7.4 |  |  | DNAj homolog subfamily a member 4 |
|  |  | **unigene22402340** |  | 3.7 |  |  | Zinc transporter zip1 |
|  |  | unigene22387546 |  | 4.6 |  |  | Peptidoglycan-recognition protein sc2-like |
|  |  | unigene22396210 |  | 4.5 |  |  | P2y purinoceptor 13 |
|  |  | **unigene22393907** |  | 11.5 |  |  | C-X-C motif chemokine 11-like |
|  |  | unigene22401785 |  | 3.8 |  |  | Heme oxygenase-like |
|  |  | **unigene22396352** |  | 4.0 |  |  | P-selectin precursor |
|  |  | unigene22398574 |  | 5.2 |  |  | Marcks-related |
|  |  | unigene22389559 |  | 3.4 |  |  | Deleted in malignant brain tumors 1 |
|  |  | **unigene22387334** |  | 3.6 |  |  | Collagenase 3 precursor |
|  |  | unigene22386429 |  | 6.5 |  |  | Unnamed protein product |
|  |  | **unigene22394184** |  |  |  | 3.9 | Syndecan-4 isoform x2 |
|  |  | **unigene22400989** |  | 4.5 |  |  | Cholesterol 25-hydroxylase-like protein |
|  |  | unigene22393889 |  | 4.9 |  |  | Serine threonine-protein kinase pim-2-like |
|  |  | **unigene22401072** |  | 3.4 |  |  | 78 kDa glucose-regulated protein precursor |
|  |  | **unigene22397165** |  | 4.1 |  |  | Interferon-induced protein 44-like |
|  |  | unigene22387202 |  | 6.1 |  |  | Tnfaip3-interacting protein 1-like |
|  |  | unigene22395999 |  | 3.8 |  |  | Mitogen-activated protein kinase 4-like |
|  |  | unigene22394805 |  |  |  | 3.7 | UDP-glucuronosyltransferase 2b31-like |
|  |  | unigene22395911 |  | 3.5 |  |  | Polymeric immunoglobulin receptor-like protein |
|  | Liver | **unigene22386313** | 41.7 |  |  |  | L-serine dehydratase l-threonine deaminase |
|  |  | **unigene22387334** |  | 11.5 |  |  | Collagenase 3 precursor |
|  |  | unigene22389353 |  | 29.7 |  | 26.2 | Toll-like receptor 5 |
|  |  | unigene22390242 |  |  |  | 7.3 | Preli domain-containing protein mitochondrial-like |
|  |  | unigene22390752 |  |  | 130.1 |  | NA |
|  |  | unigene22391480 |  | 16.3 | 11.9 | 8.3 | Transmembrane protein 116 |
|  |  | **unigene22391652** |  |  | 22.8 |  | Matrix metalloproteinase-19-like |
|  |  | **unigene22391669** | 4.7 |  |  |  | Solute carrier family facilitated glucose transporter member 6 |
|  |  | unigene22391703 |  | 7.2 |  | 8.5 | Complement component c7-like |
|  |  | unigene22391908 |  | 11.1 |  | 8.0 | Alpha-1-acid glycoprotein 1-like |
|  |  | unigene22391932 |  | 7.2 |  | 4.9 | Mannose-1-phosphate guanyltransferase beta |
|  |  | unigene22391967 |  | 3.7 |  | 5.0 | Antigen peptide transporter 1-like |
|  |  | unigene22392531 |  | 8.8 |  |  | Microfibril-associated glycoprotein 4-like |
|  |  | unigene22392584 |  |  |  | -22.6 | Maguk p55 subfamily member 4-like |
|  |  | unigene22392836 |  | 23.9 |  | 18.9 | Toll-like receptor 5 |
|  |  | **unigene22393641** | 10.9 | 19.7 |  |  | Claudin-1 |
|  |  | **unigene22394184** |  | 10.2 |  | 8.3 | Syndecan-4 isoform x2 |
|  |  | unigene22394694 |  | 14.2 |  |  | Map kinase-interacting serine threonine-protein kinase 1-like |
|  |  | **unigene22396352** |  | 13.7 | 7.2 | 13.3 | P-selectin precursor |
|  |  | **unigene22396514** | 7.7 |  |  | 184.8 | Interleukin-1 receptor type II |
|  |  | unigene22396803 |  | 12.7 | 11.6 | 6.5 | CD59 glyco |
|  |  | unigene22396918 |  | 8.0 | 4.9 | 8.3 | Chloride intracellular channel protein 2 |
|  |  | unigene22396940 |  | 14.5 |  |  | Centromere protein j-like |
|  |  | **unigene22397018** | 64.7 |  |  |  | Cis-aconitate decarboxylase-like |
|  |  | **unigene22397285** | 11.4 | 27.0 | 17.6 |  | Serum amyloid a protein precursor |
|  |  | unigene22397731 |  | 5.5 |  | 6.0 | Glucosamine 6-phosphate n-acetyltransferase |
|  |  | unigene22398079 |  | 3.8 |  | 4.4 | NA |
|  |  | **unigene22398493** |  |  |  | -37.5 | C-C motif chemokine 13 precursor |
|  |  | **unigene22398806** |  | 13.3 |  |  | Interleukin-17 receptor a-like |
|  |  | unigene22399171 |  | -6.6 | -3.8 | -4.5 | Diacylglycerol o-acyltransferase 2 |
|  |  | **unigene22400068** | 9.7 |  |  |  | Complement c1q tumor necrosis factor-related protein 3-like |
|  |  | **unigene22400741** | 6.3 |  | 6.3 |  | Metalloreductase steap4 |
|  |  | **unigene22401072** |  | 19.4 |  | 9.6 | 78 kDa glucose-regulated protein precursor |
|  |  | unigene22401107 |  | 5.4 |  | 7.5 | 3-methyl-2-oxobutanoate dehydrogenase |
|  |  | unigene22401110 |  | 34.0 |  |  | Lipid phosphate phosphohydrolase 1-like |
|  |  | unigene22401273 |  | -7.3 | -4.8 | -7.4 | Regulator of microtubule dynamics protein 2 |
|  |  | unigene22401769 |  | -3.6 |  | -6.1 | Protein phosphatase 1 regulatory subunit 3c-b-like |
|  |  | **unigene22402340** |  | 13.0 | 4.2 | 10.0 | Zinc transporter zip1 |
|  |  | unigene22402438 |  | 14.4 |  |  | Sarcoplasmic endoplasmic reticulum calcium ATPase 2 isoform x1 |
|  |  | unigene22403494 |  | 81.7 |  |  | Leptin |
|  |  | unigene22403774 |  | -9.4 |  |  | Sam domain-containing protein samsn-1 |
|  |  | **unigene22404077** | 58.7 | 55.7 | 61.5 | 40.5 | Cathelicidin antimicrobial peptide precursor |
|  |  | unigene22404382 |  | 4.3 | 3.4 | 4.4 | Ubiquitin-conjugating enzyme e2 d2 |
|  |  | **unigene22404772** |  | 8.6 | 10.0 | 7.4 | 25-hydroxycholesterol 7-alpha-hydroxylase-like |
|  |  | unigene22404773 |  | 35.6 | 20.0 |  | Class e basic helix-loop-helix protein 22-like |
|  |  | **unigene22405217** | 4.0 | 7.6 | 7.2 |  | C type lectin receptor b |
|  |  | **unigene22405218** | 5.7 |  | 7.9 |  | CD209 antigen-like protein e |
|  |  | unigene22406189 |  | 18.9 |  |  | Lim domain and actin-binding protein 1-like |
|  |  | **unigene22406570** | 6.0 | 8.9 |  | 5.4 | Nucleobindin-2-like |
|  |  | **unigene22406621** |  | 10.1 | 8.6 | 11.1 | Interleukin-17 receptor a-like |
|  |  | unigene22407014 |  |  |  | 6.7 | Suppressor of swi4 1 homolog |
|  |  | unigene22407119 |  | 7.1 |  | 6.8 | SH2 domain-containing protein 4a-like |
|  |  | unigene22410915 |  | 8.0 |  | 5.4 | DNAj homolog subfamily b member 9-like |
|  |  | unigene22412505 |  | -5.0 |  | -5.3 | NA |
|  |  | unigene22414287 |  | 7.4 |  | 5.4 | Serine--tRNA cytoplasmic |
|  |  | unigene22414636 |  |  |  | 17.2 | Proline dehydrogenase mitochondrial-like |
|  |  | unigene22414753 |  | 18.3 |  | 10.2 | Golgi-associated pdz and coiled-coil motif-containing protein isoform x1 |
|  |  | unigene22414754 |  | 14.5 |  |  | Golgi-associated pdz and coiled-coil motif-containing protein isoform x1 |
|  |  | unigene22414923 |  | 4.8 |  | 5.6 | Eukaryotic peptide chain release factor gtp-binding subunit ERF3b |
|  |  | **unigene22415017** |  | 9.6 |  |  | Suppressor of cytokine signaling 3-like |
|  |  | unigene22415254 |  | 7.1 |  | 5.5 | ATP-binding cassette sub-family f member 2 |
|  |  | **unigene22415255** |  | 7.6 |  | 5.2 | ATP-binding cassette sub-family f member 2 |
|  |  | unigene22415583 |  | 11.1 | 5.8 | 13.8 | Intercellular adhesion molecule 3 isoform x2 |
|  |  | unigene22415619 |  | 4.3 |  | 4.0 | Nop58 protein |
|  |  | unigene22416115 |  | 4.2 |  | 5.8 | Junctional adhesion molecule b isoform x1 |
|  |  | unigene22416286 |  |  | 4.1 |  | Tnfaip3-interacting protein 1 isoform x1 |
|  |  | unigene22416287 |  | 3.8 | 4.3 |  | Tnfaip3-interacting protein 1 |
|  |  | unigene22417188 |  | -5.3 |  |  | Cytochrome p450 1a |
|  |  | unigene22418518 |  | 7.1 |  | 6.0 | Sugar phosphate exchanger 2 |
|  |  | **unigene22419056** |  | 7.9 |  | 7.2 | Adenosine monophosphate-protein transferase ficd |
|  |  | unigene22419231 |  |  | -3.9 |  | Pyruvate dehydrogenase (acetyl-transferring) kinase isozyme mitochondrial-like |
|  |  | unigene22420325 |  | 5.0 |  |  | Toll-like receptor tlr22a2 |
|  |  | unigene22420783 |  | 8.6 |  |  | ATP-binding cassette sub-family f member 3 |
|  |  | **unigene22421222** |  | -12.0 | -7.5 | -5.7 | Diablo mitochondrial |
|  |  | unigene22421223 |  | -9.9 |  | -5.5 | Diablo mitochondrial |
|  |  | **unigene22422936** |  | 9.8 |  |  | Matrix metalloproteinase-9 |
|  |  | unigene22423893 |  | 3.8 | 3.1 |  | Eukaryotic translation initiation factor 4 gamma 2 |
|  |  | unigene22423894 |  | 3.7 |  |  | Eukaryotic translation initiation factor 4 gamma 2 |
|  |  | unigene22423984 |  | 4.9 |  | 5.5 | Coatomer subunit beta |
|  |  | unigene22424248 |  | 3.9 |  | 3.8 | NA |
|  |  | **unigene22426055** | 25.5 | 7.3 |  |  | Protein dopey-1 |
|  |  | **unigene22426377** |  | 17.0 |  | 24.4 | NA |
|  |  | unigene22425976 |  |  | 5.4 |  | NA |
|  |  | unigene22422468 |  | 3.8 |  | 3.8 | Signal recognition particle 14 kDa protein |
|  |  | unigene22417210 |  | 4.8 |  |  | Ras GTPase-activating protein-binding protein 2 |
|  |  | unigene22413143 |  | 5.9 |  | 3.7 | DNAj homolog subfamily c member 1 |
|  |  | unigene22417259 |  | 5.7 |  | 5.0 | Importin subunit alpha-2 |
|  |  | unigene22413730 |  | 9.7 |  |  | Heat shock 70 kDa protein 13 |
|  |  | unigene22417489 |  |  |  | 4.3 | Muscarinic acetylcholine receptor m5-like |
|  |  | unigene22421785 |  | 5.5 |  |  | Heterogeneous nuclear ribonucleoprotein |
|  |  | unigene22414354 |  | 4.8 |  | 3.3 | Ubiquitin-like modifier-activating enzyme 5 |
|  |  | unigene22426266 |  |  | 4.6 |  | Saxitoxin and tetrodotoxin-binding protein 1 |
|  |  | unigene22409750 |  |  |  | 3.7 | Calumenin-a |
|  |  | unigene22407487 |  | 42.2 |  |  | Interleukin-1 receptor accessory |
|  |  | unigene22409596 |  | 9.0 |  |  | Guanine nucleotide-binding protein g subunit alpha |
|  |  | unigene22406042 |  |  |  | 3.1 | AP-1 complex subunit mu-2 |
|  |  | unigene22404438 |  | -4.3 |  |  | Abhydrolase domain-containing protein 15-like |
|  |  | unigene22410328 |  | 3.9 |  |  | Tumor necrosis factor receptor superfamily member 6b |
|  |  | unigene22407580 |  | 10.9 |  |  | Hypoxia up-regulated protein 1 |
|  |  | unigene22409607 |  | 5.1 |  |  | Transmembrane protein 208-like |
|  |  | unigene22411563 |  | 3.8 |  | 4.1 | Protein transport protein sec24c |
|  |  | unigene22409781 |  | 3.4 |  | 3.9 | Ras-related protein rab-1a |
|  |  | unigene22404365 |  |  | 5.9 |  | NF-kappa-b inhibitor alpha |
|  |  | **unigene22410673** |  |  |  | 4.3 | Peptidyl-prolyl cis-trans isomerase fkbp5-like |
|  |  | unigene22411523 |  | 5.6 |  | 4.2 | Alpha- -mannosyl-glycoprotein 2-beta-n-acetylglucosaminyltransferase-like |
|  |  | unigene22406447 |  |  |  | 4.8 | Thioredoxin domain-containing protein 11 |
|  |  | unigene22401912 |  | -12.4 |  |  | Inhibin beta b chain-like |
|  |  | unigene22401073 |  | 10.7 |  |  | 78 kDa glucose-regulated protein precursor |
|  |  | unigene22400778 |  | 4.4 |  | 4.5 | DNAj homolog subfamily b member 9-like |
|  |  | unigene22396326 |  | 5.2 |  | 4.2 | Tubulin alpha-1b partial |
|  |  | unigene22391966 |  | 3.7 |  | 4.9 | Transporter-associated with antigen processing 1 |
|  |  | unigene22396644 |  | 13.7 |  |  | ATPase Ca++ transporting cardiac muscle slow twitch 2 |
|  |  | unigene22395533 |  |  |  | 20.0 | Vesicle transport protein got1b |
|  |  | unigene22391878 |  | 6.8 |  |  | Mki67 fha domain-interacting nucleolar phosphoprotein |
|  |  | unigene22396887 |  | 4.2 |  | 3.8 | Tubulin alpha-1c chain- partial |
|  |  | unigene22399279 |  | 20.5 |  |  | Hemicentin-1-like isoform x2 |
|  |  | unigene22400098 |  | 3.9 |  |  | Tubulin alpha-4a partial |
|  |  | unigene22393604 |  | -4.0 |  |  | Ino80 complex subunit c |
|  |  | unigene22397558 |  |  | 7.5 |  | Marcks-related protein |
|  |  | unigene22390234 |  | 4.1 |  | 4.0 | Surfeit locus protein 6 |
|  |  | unigene22395557 |  | 16.8 |  | 8.0 | Mesencephalic astrocyte-derived neurotrophic factor |
|  |  | unigene22386664 |  | -6.4 |  |  | Fibroblast growth factor 19 |
|  |  | unigene22395192 |  |  |  | 6.0 | Apoptosis-enhancing nuclease |
|  |  | unigene22421786 |  | 5.0 |  |  | NA |
|  |  | unigene22415584 |  | 25.0 |  |  | Intercellular adhesion molecule 5 isoform x1 |
|  |  | unigene22423878 |  |  | 4.5 |  | Nuclear factor NF-kappa-b p100 subunit factor NF-kappa-b p52 subunit |
|  |  | unigene22426252 |  |  |  | 3.6 | Ribosome biogenesis protein brx1 homolog |
|  |  | unigene22426289 |  | 3.3 |  |  | NA |
|  |  | unigene22422419 |  |  |  | 3.1 | Probable ATP-dependent RNA helicase ddx5 |
|  |  | unigene22420294 |  | 7.5 |  |  | Eukaryotic translation initiation factor 4 gamma 1-like isoform x3 |
|  |  | unigene22414081 |  | 3.3 |  |  | Protein arginine n-methyltransferase 1 |
|  |  | unigene22425478 |  | 3.4 |  |  | Cullin-9 |
|  |  | unigene22414186 |  |  |  | 4.0 | H aca ribonucleoprotein complex subunit 4 |
|  |  | unigene22403628 |  | -3.7 |  |  | CD80-like protein |
|  |  | unigene22404383 |  |  |  | 4.0 | Ubiquitin-conjugating enzyme e2 d2 |
|  |  | unigene22405504 |  |  | -3.9 |  | Gsk-3-binding protein |
|  |  | unigene22402684 |  | -4.0 |  |  | Nuclear receptor subfamily 0 group b member 2 |
|  |  | unigene22403801 |  | 3.9 |  |  | Activator of 90 kDa heat shock protein ATPase homolog 1-like |
|  |  | unigene22407728 |  |  |  | 4.1 | Tho complex subunit 4 |
|  |  | unigene22408185 |  | 9.4 |  |  | Sarcoplasmic endoplasmic reticulum calcium ATPase 2-like isoform x2 |
|  |  | unigene22401862 |  | 52.9 |  |  | Growth arrest and DNA-damage-inducible protein gadd45 beta |
|  |  | unigene22402830 |  | 6.1 |  |  | Ubiquitin carboxyl-terminal hydrolase 16 |
|  |  | unigene22411536 |  |  |  | 3.4 | Lyric protein |
|  |  | unigene22409521 |  | 6.6 |  |  | Ornithine decarboxylase |
|  |  | unigene22409522 |  | 7.6 |  |  | Ornithine decarboxylase |
|  |  | unigene22409531 |  |  | 4.2 |  | Anosmin-1-like isoform x1 |
|  |  | **unigene22407218** |  | 11.5 |  |  | DNAj homolog subfamily b member 11-like |
|  |  | unigene22392119 |  | 5.2 |  |  | ER lumen protein retaining receptor 2-like |
|  |  | unigene22387531 |  | 5.5 |  |  | Ifngr2-related protein |
|  |  | unigene22393846 |  |  |  | 3.4 | Probable ATP-dependent RNA helicase ddx27-like |
|  |  | unigene22394174 |  | 3.2 |  |  | Calcium-regulated heat stable protein 1 |
|  |  | **unigene22390428** |  |  |  | 3.9 | DNAj homolog subfamily c member 3 |
|  |  | **unigene22395558** |  |  |  | 5.6 | Mesencephalic astrocyte-derived neurotrophic factor-like |
|  |  | unigene22391038 |  | 5.1 |  |  | Ribosomal l1 domain-containing protein 1 |
|  |  | **unigene22387109** |  | 3.7 |  |  | Unnamed protein product |
|  |  | **unigene22397165** | 3.0 |  |  |  | Interferon-induced protein 44-like |
|  |  | unigene22397452 |  | 4.7 |  |  | Ribosomal RNA processing protein 1 homolog b-like isoform x1 |
|  |  | unigene22397346 |  | 5.7 |  |  | Alpha- -mannosyltransferase alg2 |
|  |  | unigene22415902 |  |  |  | 3.1 | Solute carrier family 35 member b1 |
|  |  | unigene22424893 |  |  |  | 4.2 | Cytochrome c |
|  |  | unigene22426438 |  | 3.9 |  |  | NA |
|  |  | unigene22408187 |  | 12.5 |  |  | Sarcoplasmic endoplasmic reticulum calcium ATPase 2 isoform x3 |
|  |  | unigene22408217 |  |  | 4.3 |  | NADPh oxidase organizer 1 |
|  |  | unigene22402125 |  | 5.3 |  |  | Beta tubulin |
|  |  | unigene22396325 |  | 3.6 |  |  | Tubulin alpha-4a chain |
|  |  | unigene22393737 |  | 11.8 |  |  | Unnamed protein product |
|  |  | unigene22390291 |  |  |  | 3.3 | Protein sec13 homolog |
| Poly I:C | Head | **unigene22416457** |  | 25.0 |  | 11.1 | Interferon-induced gtp-binding protein mx-like |
|  | kidney | **unigene22417416** |  | 3.4 |  | 5.4 | Helicase with zinc finger domain 2 |
|  |  | unigene22416724 |  | 4.5 |  |  | Poly polymerase 12 |
|  |  | **unigene22390277** |  | 7.8 |  |  | NA |
|  |  | unigene22411063 |  | 3.4 |  | 4.0 | Tripartite motif-containing protein 16 |
|  |  | **unigene22396321** |  | 7.4 |  |  | Down syndrome cell adhesion molecule-like protein dscam2-like |
|  |  | unigene22416359 |  | 7.8 |  |  | Signal transducer and activator of transcription 1-alpha beta isoform x1 |
|  |  | unigene22406229 |  | 4.4 |  |  | Ring finger protein 135 |
|  |  | unigene22422100 |  | 9.3 |  |  | NA |
|  |  | **unigene22411130** |  | 6.8 |  |  | Eukaryotic translation initiation factor 4 gamma 3- partial |
|  |  | **unigene22392264** |  | 4.3 |  |  | Signal transducer and activator of transcription 2 isoform x1 |
|  |  | unigene22425227 |  | 5.0 |  |  | Stonustoxin subunit alpha |
|  |  | unigene22392766 |  | 4.5 |  |  | Interferon-inducible protein gig2-like |
|  |  | unigene22409187 |  | 4.4 |  |  | CD9 antigen |
|  |  | unigene22392975 |  | 6.1 |  |  | Tripartite motif-containing protein 47-like isoform x2 |
|  |  | unigene22420410 |  | 13.5 |  |  | Nfx1-type zinc finger-containing protein 1-like |
|  |  | **unigene22394288** |  | 4.8 |  |  | E3 ubiquitin-protein ligase rnf213-like |
|  |  | **unigene22394538** |  | 4.5 |  |  | Opioid growth factor receptor |
|  |  | unigene22385616 |  | 5.2 |  |  | Unnamed protein product |
|  |  | **unigene22394665** |  | 3.6 |  |  | Interferon-induced helicase c domain-containing protein 1-like |
|  |  | unigene22406766 |  | 6.0 |  |  | Protein kinase containing z-DNA binding domains |
|  |  | unigene22394920 |  | 6.5 |  |  | Unnamed protein product |
|  |  | unigene22410971 |  | 3.3 |  |  | Tripartite motif-containing protein 39 |
|  |  | unigene22395092 |  | 4.2 |  |  | Polyadenylate-binding protein 1 |
|  |  | unigene22411959 |  | 4.5 |  |  | Sub-family b ATP-binding cassette transporter 2 |
|  |  | unigene22395308 |  | 10.4 |  |  | Lrr and pyd domains-containing protein 3- partial |
|  |  | **unigene22420791** |  | 4.6 |  |  | Signal transducer and activator of transcription 1 |
|  |  | unigene22395924 |  | 5.7 |  |  | Zinc-binding protein a33-like |
|  |  | unigene22422890 |  | 3.4 |  |  | 0 |
|  |  | **unigene22395942** |  | 9.1 |  |  | Suppressor of cytokine signaling 1 |
|  |  | unigene22422988 |  | 4.3 |  |  | E3 ubiquitin-protein ligase rnf213 |
|  |  | unigene22386451 |  | 13.5 |  |  | Reproduction regulator 2 |
|  |  | unigene22405102 |  | 41.3 |  |  | Platelet basic protein precursor |
|  |  | unigene22396660 |  | 8.6 |  |  | Probable e3 ubiquitin-protein ligase rnf144a-a |
|  |  | unigene22406033 |  |  |  | 9.9 | Sterile alpha motif domain-containing protein 9-like |
|  |  | unigene22396661 |  | 10.5 |  |  | Probable e3 ubiquitin-protein ligase rnf144a-a |
|  |  | unigene22406582 |  | 8.3 |  |  | C-C motif chemokine 19 precursor |
|  |  | unigene22397125 |  | 5.8 |  |  | Xiap-associated factor 1 |
|  |  | unigene22408013 |  | 4.5 |  |  | Unnamed protein product |
|  |  | unigene22397126 |  | 9.6 |  |  | Xiap-associated factor 1 |
|  |  | unigene22410436 |  | 5.8 |  |  | Probable gpi-anchored adhesin-like protein pga55 |
|  |  | unigene22413158 |  | 23.1 |  |  | Probable e3 ubiquitin-protein ligase herc4-like |
|  |  | unigene22387305 |  | 21.7 |  |  | Sacsin-like |
|  |  | unigene22386861 |  | 4.8 |  |  | Poly(ADP-ribose) glycohydrolase-like |
|  |  | unigene22411579 |  | 5.9 |  |  | Zinc finger ccch-type antiviral protein 1 |
|  |  | unigene22385938 |  | 10.4 |  |  | Zinc-binding protein a33-like |
|  |  | **unigene22387109** |  | 4.7 |  |  | Unnamed protein product |
|  |  | **unigene22397165** |  | 4.8 |  |  | Interferon-induced protein 44-like |
|  |  | unigene22416703 |  | 7.4 |  |  | PREDICTED: uncharacterized protein LOC103395660 |
|  |  | **unigene22397431** |  | 3.0 |  |  | Helicase mov-10- -like |
|  |  | unigene22417415 |  | 6.4 |  |  | Helicase with zinc finger domain 2-like |
|  |  | unigene22420071 |  | 6.6 |  |  | DNA damage-regulated autophagy modulator protein 1 |
|  |  | unigene22397960 |  | 3.5 |  |  | Poly |
|  |  | **unigene22420471** |  | 3.6 |  |  | Gig2-like protein |
|  |  | unigene22398548 |  | 4.1 |  |  | Uridine-cytidine kinase-like 1-like isoform x2 |
|  |  | unigene22401815 |  | 17.5 |  |  | Nfx1-type zinc finger-containing protein 1 |
|  |  | unigene22422886 |  | 5.0 |  |  | 0 |
|  |  | unigene22401816 |  | 5.5 |  |  | Nfx1-type zinc finger-containing protein 1 |
|  |  | unigene22422986 |  | 6.9 |  |  | E3 ubiquitin-protein ligase rnf213 |
|  |  | **unigene22403329** |  | 4.7 |  |  | Unnamed protein product |
|  |  | unigene22423922 |  | 4.5 |  |  | Stonustoxin subunit beta |
|  |  | unigene22404503 |  | 7.7 |  |  | Vig- protein |
|  |  | unigene22412822 |  | 3.0 |  |  | Tyrosine-protein kinase frk isoform x2 |
|  |  | unigene22399760 |  | 3.7 |  |  | Plasminogen activator inhibitor 1 |
|  |  | unigene22399845 |  | 3.0 |  |  | Poly(ADP-ribose) glycohydrolase-like |
|  |  | **unigene22400989** |  | 3.7 |  |  | Cholesterol 25-hydroxylase-like protein |
|  |  | **unigene22401072** |  | 3.1 |  |  | 78 kDa glucose-regulated protein precursor |
|  |  | unigene22399757 |  | 3.4 |  |  | E3 ubiquitin-protein ligase rnf8 |
|  |  | unigene22401363 |  | 4.3 |  |  | Nuclear receptor coactivator 7-like |
|  |  | **unigene22392798** |  | 4.4 |  |  | C-type lectin domain family 4 member m-like isoform x1 |
|  |  | unigene22391337 |  |  |  | 5.8 | E3 ubiquitin-protein ligase rnf213-like |
|  |  | unigene22401962 |  | 3.6 |  |  | Periphilin-1 |
|  |  | unigene22422891 |  |  |  | 4.5 | Sterile alpha motif domain-containing protein 9-like |
|  |  | unigene22393778 |  | 3.5 |  |  | Mitochondrial ubiquitin ligase activator of nfkb 1-a-like |
|  |  | unigene22416481 |  | 3.2 |  |  | Receptor-interacting serine threonine-protein kinase 3-like |
|  |  | unigene22416669 |  | 4.4 |  |  | NA |
|  |  | **unigene22393907** |  | 12.6 |  |  | C-X-C motif chemokine 11-like |
|  |  | unigene22404160 |  | 8.8 |  |  | Apolipoprotein l3-like isoform x2 |
|  |  | **unigene22391779** |  | 4.0 |  |  | Interferon-induced helicase c domain-containing protein 1-like |
|  |  | unigene22399714 |  | 14.1 |  |  | Poly |
|  |  | **unigene22426238** |  | 5.9 |  |  | NA |
|  |  | **unigene22395558** |  | 3.2 |  |  | Mesencephalic astrocyte-derived neurotrophic factor-like |
|  |  | unigene22394082 |  | 3.9 |  |  | Unnamed protein product |
|  |  | **unigene22405479** |  | 3.0 |  |  | Nicotinamide phosphoribosyltransferase |
|  |  | unigene22416195 |  | 3.1 |  |  | Deoxycytidine kinase |
|  |  | unigene22394181 |  | 3.4 |  |  | Tapasin precursor |
|  |  | unigene22406034 |  | 8.3 |  |  | Sterile alpha motif domain-containing protein 9-like |
|  |  | unigene22386258 |  | 4.6 |  |  | Gig2-like protein |
|  |  | unigene22387135 |  | 3.6 |  |  | Regulator of cell cycle rgcc-like |
|  |  | unigene22397082 |  |  |  | 8.6 | GTPase imap family member 7-like |
|  |  | **unigene22418322** |  | 3.1 |  |  | E3 ubiquitin-protein ligase trim21-like |
|  |  | unigene22406767 |  | 6.7 |  |  | Z-DNA binding protein kinase |
|  |  | unigene22418765 |  | 3.9 |  |  | Insulin-induced gene 1 protein |
|  |  | **unigene22407218** |  | 3.2 |  |  | DNAj homolog subfamily b member 11-like |
|  |  | unigene22409426 |  | 3.4 |  |  | GTPase imap family member 7-like |
|  |  | unigene22398766 |  | 3.0 |  |  | PREDICTED: uncharacterized protein LOC105022384 isoform X2 |
|  |  | unigene22423925 |  | 4.1 |  |  | E3 ubiquitin isg15 ligase trim25conjugating enzyme trim25 |
|  |  | unigene22395265 |  | 3.6 |  |  | Polymeric immunoglobulin receptor |
|  |  | **unigene22411131** |  | 8.2 |  |  | Eukaryotic translation initiation factor 4 gamma 3- partial |
|  |  | unigene22404819 |  | 3.5 |  |  | E3 ubiquitin isg15 ligase trim25-like |
|  |  | unigene22404821 |  | 8.1 |  |  | E3 ubiquitin isg15 ligase trim25-like isoform x4 |
|  | Liver | **unigene22394665** |  | 6.4 |  | 4.8 | Interferon-induced helicase c domain-containing protein 1-like |
|  |  | **unigene22411130** |  | 7.8 |  | 4.7 | Eukaryotic translation initiation factor 4 gamma 3- partial |
|  |  | **unigene22398806** |  | 6.1 |  | 5.9 | Interleukin-17 receptor a-like |
|  |  | unigene22409186 |  | 14.7 |  | 9.8 | CD9 antigen |
|  |  | **unigene22403329** |  | 9.3 |  | 9.1 | Unnamed protein product |
|  |  | unigene22416725 |  | 4.8 |  | 3.7 | Poly polymerase 12 |
|  |  | **unigene22391779** |  |  |  | 4.7 | Interferon-induced helicase c domain-containing protein 1-like |
|  |  | **unigene22406621** |  |  |  | 5.8 | Interleukin-17 receptor a-like |
|  |  | unigene22392306 |  | 31.5 |  | 13.8 | Cat eye syndrome critical region protein 5 homolog |
|  |  | **unigene22411131** |  | 15.7 |  | 6.6 | Eukaryotic translation initiation factor 4 gamma 3- partial |
|  |  | unigene22392883 |  | 14.7 |  |  | DsRNA-activated protein kinase r |
|  |  | **unigene22390428** |  | 3.9 |  | 4.3 | DNAj homolog subfamily c member 3 |
|  |  | **unigene22394538** |  | 6.7 |  | 4.8 | Opioid growth factor receptor |
|  |  | unigene22422654 |  | 11.7 |  |  | Poly |
|  |  | unigene22385855 |  | 11.8 |  | 16.4 | Interferon-induced very large GTPase 1-like |
|  |  | **unigene22407218** |  | 8.9 |  | 5.5 | DNAj homolog subfamily b member 11-like |
|  |  | unigene22394666 |  |  |  | 4.3 | Interferon-induced helicase c domain-containing protein 1-like |
|  |  | **unigene22387109** |  | 5.1 |  | 4.5 | Unnamed protein product |
|  |  | **unigene22397431** |  | 4.5 |  | 3.2 | Helicase mov-10- -like |
|  |  | **unigene22415255** |  | 4.1 |  |  | ATP-binding cassette sub-family f member 2 |
|  |  | **unigene22420791** |  | 7.5 |  |  | Signal transducer and activator of transcription 1 |
|  |  | **unigene22418322** |  | 12.1 |  |  | E3 ubiquitin-protein ligase trim21-like |
|  |  | unigene22420793 |  |  |  | 3.2 | Signal transducer and activator of transcription 3 |
|  |  | **unigene22402340** |  | 6.0 |  |  | Zinc transporter zip1 |
|  |  | unigene22402141 |  | 20.3 |  | 10.5 | Interferon regulatory factor 7 |
|  |  | **unigene22405479** |  | 4.3 |  | 4.3 | Nicotinamide phosphoribosyltransferase |
|  |  | **unigene22392264** |  |  |  | 6.5 | Signal transducer and activator of transcription 2 isoform x1 |
|  |  | unigene22418951 |  |  |  | 6.3 | Poly |
|  |  | unigene22387611 |  |  |  | 24.7 | Sacsin-like isoform x1 |
|  |  | unigene22391050 |  |  |  | 3.3 | Galectin-3-binding protein precursor |
|  |  | unigene22419415 |  |  |  | 7.7 | Nipped-b-like partial |
|  |  | **unigene22421222** |  | -3.8 |  |  | Diablo mitochondrial |
|  |  | unigene22409038 |  |  |  | 5.4 | Microtubule-associated protein rp eb family member 2 isoform x1 |
|  |  | unigene22398482 |  |  |  | 9.1 | UMP-CMP kinase mitochondrial |
|  |  | **unigene22417416** |  |  |  | 9.6 | Helicase with zinc finger domain 2 |
|  |  | **unigene22419056** |  |  |  | 6.3 | Adenosine monophosphate-protein transferase ficd |
|  |  | **unigene22394288** |  |  |  | 7.0 | E3 ubiquitin-protein ligase rnf213-like |
|  |  | **unigene22420471** |  |  |  | 8.0 | Gig2-like protein |
|  |  | **unigene22401072** |  |  |  | 7.7 | 78 kDa glucose-regulated protein precursor |
|  |  | unigene22413680 |  | 10.0 |  |  | GTPase imap family member 7-like |
|  |  | **unigene22395942** |  |  |  | 9.8 | Suppressor of cytokine signaling 1 |
|  |  | **unigene22390277** |  |  |  | 7.4 | NA |
|  |  | unigene22422965 |  |  |  | 12.6 | Probable e3 ubiquitin-protein ligase herc3 |
|  |  | **unigene22426377** |  | 7.2 |  |  | NA |
|  |  | unigene22426360 |  |  |  | 10.9 | NA |
|  |  | unigene22399284 | 7.3 |  |  |  | Heat shock protein hsp 90-alpha |

PBS, phosphate-buffered saline; PGN, peptidoglycan; Poly I:C, polyinosinic-polycytidylic acid. W, non-transgenic (wild-type) coho salmon on a full satiation ration; TF, GH transgenic coho salmon on a full satiation ration; TR, GH transgenic coho salmon on restricted ration equal to that consumed by W; D, domesticated coho salmon on a full satiation ration.
