# Supplementary material for: Effect of growth rate on transcriptomic responses to immune stimulation in wild-type, domesticated, and GH-transgenic coho salmon
Source: BMC Genomics. 2019 Dec 27;20:1024. doi: 10.1186/s12864-019-6408-4 (PMC6935076; doi:10.1186/s12864-019-6408-4)
Supplement: Supplementary file 2 — Additional file 2: Table S2. Raw reads of each group, tissue, and treatment. [file 12864_2019_6408_MOESM2_ESM.docx]

Table S2. Raw reads of each group, tissue, and treatment.

| **Treatment** | **Group** | **Head kidney** | **Liver** |
| --- | --- | --- | --- |
| PBS | D | 11,781,759 | 11,691,536 |
|  | W | 11,599,756 | 12,068,193 |
|  | TF | 12,229,682 | 11,620,779 |
|  | TR | 12,273,527 | 11,710,644 |
| PGN | D | 11,646,824 | 12,090,545 |
|  | W | 11,041,351 | 11,546,412 |
|  | TF | 11,511,852 | 11,841,116 |
|  | TR | 11,400,129 | 12,099,830 |
| Poly I:C | D | 10,724,110 | 11,876,316 |
|  | W | 11,476,109 | 12,092,308 |
|  | TF | 11,260,592 | 12,302,242 |
|  | TR | 11,455,758 | 11,265,506 |
