# Supplementary material for: Effect of growth rate on transcriptomic responses to immune stimulation in wild-type, domesticated, and GH-transgenic coho salmon
Source: BMC Genomics. 2019 Dec 27;20:1024. doi: 10.1186/s12864-019-6408-4 (PMC6935076; doi:10.1186/s12864-019-6408-4)
Supplement: Supplementary file 3 — Additional file 3: Table S3. Primers and probes used in this study. [file 12864_2019_6408_MOESM3_ESM.docx]

Table S3. Primers and probes used in this study

| **Genes** | **Oligo name** | **Sequences (5´→3´)** |  |
| --- | --- | --- | --- |
| *Complement C* | Complement C-RT-F | GGGAAACACCAGGAAAGAG |  |
|  | Complement C-RT-R | CCGACCAATCACAATCACT |  |
| *IL-8* | IL-8-RT-F | TTACTGAGGGGATGAGTC |  |
|  | IL-8-RT-R | CCTTCTTAATGAGTCTACC |  |
| *IRG1* | IRG1-RT-F | TAATGGTGGTTGTGGTGGTG |  |
|  | IRG1-RT-R | GGGAAGGGGCAGTCAATGTA |  |
| *MHC 1a* | MHc 1a-RT-F | GAGGAGTGGAAGAACAACAA |  |
|  | MHc 1a-RT-R | AGCAATGATGACCAGGAGA |  |
| *Mx* | Mx-RT-F | TTCCTCTCCTTCTTTCTTCCTT |  |
|  | Mx-RT-R | CGTCCTAGTATTGACCTCAT |  |
| *RSAD2* | RSAD2-RT-F | TCAAACAGCGACAACTCCAA |  |
|  | RSAD2-RT-R | AAATCCCCTCTGTCCTGTAA |  |
| *SAA* | SAA-RT-F | CTCGGGGCAACTATGATG |  |
|  | SAA-RT-R | TTGGGGTCTGAATCGGTT |  |
| *TNFR-5B* | TNFR-5B-RT-F | TGTTCCCTTGTGGTTGTTG |  |
|  | TNFR-5B-RT-R | TGGTGATGGTGGTGATAGT |  |
| *β-actin* | B-actin-F | ATGGGCCAGAAAGACAGCTA |  |
|  | B-actin-R | AGCCACTCTCAGCTCGTTGT |  |
| *Ef-1a* | EF-1a-F | GGAGGCCCTAGACTCAATCC |  |
|  | EF-1a-R | CTGGAAGCTCTCCACACACA |  |
| *Ubiquitin* | Ubiquitin-F | ATGTCAAGGCCAAGATCCAG |  |
|  | Ubiquitin-R | ATAATGCCTCCACGAAGACG |  |

*IRG1*: Immune responsive gene 1; *IRG1*: Immune responsive gene 1; *IL-8*: Interleukin 8; *MHC1a*: Major histocompatibility complex class 1; *Mx*: Interferon-induced GTP-binding protein Mx1; *RSAD2*: Radical S-adenosyl methionine domain containing 2; *SAA*: Serum amyloid A; *TNFR-5B*: Tumor necrosis factor receptor 5b; B-actin: Beta actin; EF-1a: Elongation factor 1a
