# Supplementary material for: Effect of growth rate on transcriptomic responses to immune stimulation in wild-type, domesticated, and GH-transgenic coho salmon
Source: BMC Genomics. 2019 Dec 27;20:1024. doi: 10.1186/s12864-019-6408-4 (PMC6935076; doi:10.1186/s12864-019-6408-4)

Supplementary fig. 1

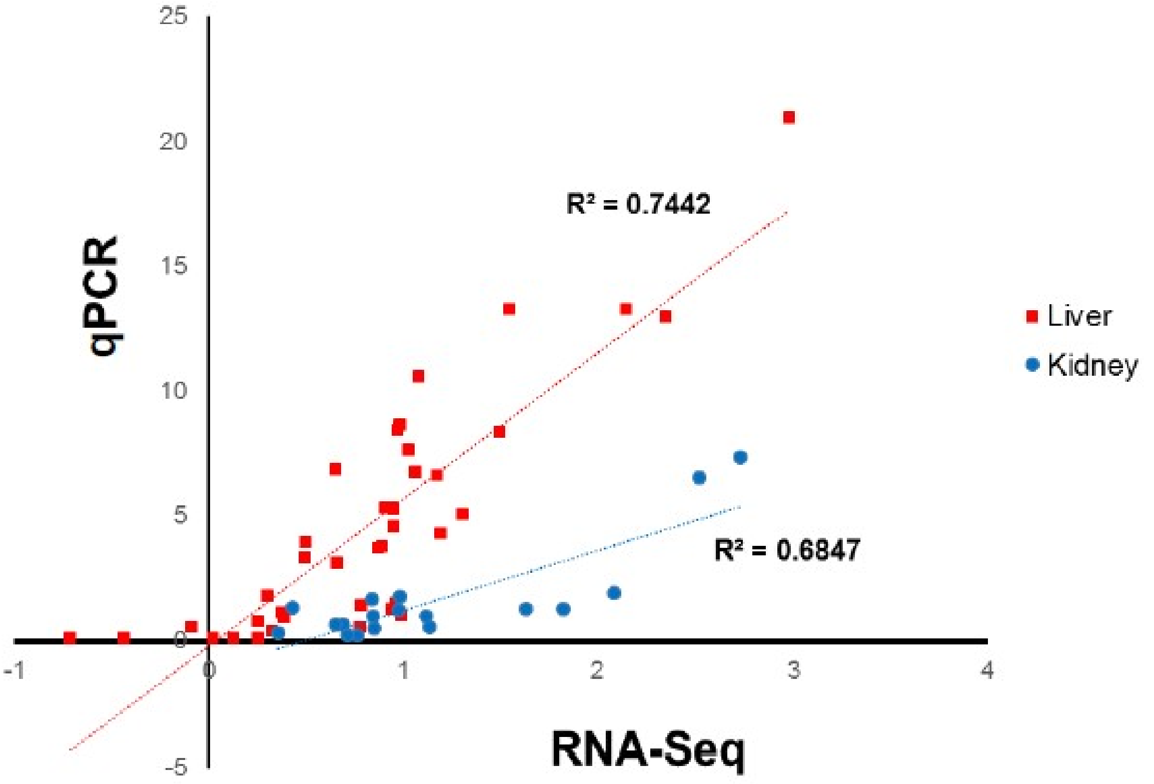

Supplement: Supplementary file 4 [file 12864_2019_6408_MOESM4_ESM.docx]
